# A lower initial dose of bosutinib for patients with chronic myeloid leukemia patients resistant and/or intolerant to prior therapy: a single-arm, multicenter, phase 2 trial (BOGI trial)

**DOI:** 10.1007/s12185-024-03830-z

**Published:** 2024-08-13

**Authors:** Hiroshi Ureshino, Naoto Takahashi, Takayuki Ikezoe, Yoshihiro Kameoka, Satoshi Kimura, Noriyasu Fukushima, Tatsuo Ichinohe, Ayako Takamori, Atsushi Kawaguchi, Masatomo Miura, Shinya Kimura

**Affiliations:** 1 Department of Hematology and Oncology, Research Institute for Radiation Biology and Medicine, Hiroshima University, Hiroshima, Japan; 2 Department of Hematology, Nephrology and Rheumatology, Akita University Graduate School of Medicine, 1‑1‑1, Hondo, Akita, Akita 010‑8543 Japan; 3 Department of Hematology, Fukushima Medical University, Fukushima, Japan; 4 Department of Internal Medicine, Karatsu Red Cross Hospital, Karatsu, Japan; 5 Clinical Research Center, Saga University Hospital, Saga, Japan; 6 Education and Research Center for Community Medicine, Faculty of Medicine, Saga University, Saga, Japan; 7 Department of Pharmacokinetics, Akita University Graduate School of Medicine, Akita, Japan; 8 Hematology, Respiratory Medicine and Oncology, Department of Internal Medicine, Saga University, Saga, Japan

**Keywords:** Chronic myeloid leukemia, Bosutinib, Tyrosine kinase inhibitor, Lower dose initiation, Pharmacokinetics

## Abstract

Although bosutinib is generally safe and effective, drug-related toxicities (DRTs) such as diarrhea or increased transaminase levels often lead to treatment discontinuation. To clarify whether a lower initial dose of bosutinib (i.e., starting at 200 mg) would reduce rates of discontinuation due to DRTs, we conducted a phase 2 study of BOsutinib Gradual Increase (BOGI trial, UMIN 000032282) as a second/third-line treatment for chronic myeloid leukemia (CML). Between February 4, 2019 and May 24, 2022, 35 patients were enrolled. The rate of bosutinib discontinuation at 12 months was 25.7% vs. 35.9% in a historical control study (Japanese phase 1/2 study) (*p* = 0.102). The rate of bosutinib discontinuation due to DRTs was significantly lower, at 11.4% vs. 28.2% (*p* = 0.015). The incidence of grade 3/4 transaminase elevation was 20% vs. 29% (*p* = 0.427), while the incidence of diarrhea was 3% vs. 25% (*p* = 0.009). The median dose intensity of bosutinib was higher (391.7 mg/day vs. 353.9 mg/day). Pharmacokinetic analysis of bosutinib showed that patients who achieved a major molecular response tended to have high trough concentrations. Thus, a low initial dose of bosutinib followed by dose escalation reduced discontinuation due to severe DRTs while maintaining high dose intensity and efficacy.

**Supplementary Information:**

The online version contains supplementary material available at 10.1007/s12185-024-03830-z.

## Introduction

The introduction of ABL1 tyrosine kinase inhibitors (TKIs) has improved survival outcomes for patients with chronic phase chronic myeloid leukemia (CML-CP) markedly [1]. Although imatinib, a pioneering ABL TKI, has many benefits for patients with CML-CP, second-generation TKIs are more effective [2, 3]. Bosutinib is a second-generation ABL TKI approved for the treatment of CML-CP resistant and/or intolerant to prior therapy, as well as for newly diagnosed patients [4–6]. Several clinical trials report the efficacy and safety of bosutinib; however, the drug is associated with drug-related toxicities (DRTs) such as diarrhea or increased transaminase levels, which often lead to treatment discontinuation [6, 7]. The standard initial daily dose of bosutinib is 400 mg for newly diagnosed patients and 500 mg for resistant/intolerant patients, although some experts recommend a lower initiating dose (200–300 mg) to reduce the incidence of adverse events (AEs) [8]; however, the safety and efficacy of lower dose initiation of bosutinib have not been investigated in detail [9]. Moreover, the association between the pharmacokinetics (PK) of low-dose bosutinib and its safety/efficacy are unclear [10, 11].

Hence, we conducted a phase 2 study of BOsutinib Gradual Increase as a second/third-line treatment for CML-CP (BOGI trial, UMIN 000032282) to ascertain whether a lower initiating dose of bosutinib (200 mg daily) would reduce discontinuation rates or treatment interruptions due to DRTs. We also investigated the PK of bosutinib at different doses.

## Methods

### Study design and patient enrollment

The BOGI trial was a single-arm, multicenter, phase 2 trial conducted across four Japanese hospitals; the aim was to investigate the safety and efficacy of low-dose initiation of bosutinib therapy for patients with CML-CP that was resistant and/or intolerant to prior therapy. Those included were CML-CP patients who developed at least one line of treatment failure (resistant and/or intolerant), were aged ≧18 years, had an Eastern Cooperative Oncology Group performance status score of 0–2, and had adequate organ function (lung, liver, and renal function parameters within normal ranges). Patients newly diagnosed with CML-CP, those with accelerated phase CML or blast phase CML, those who received any other chemotherapy (except hydroxyurea), those with a T315I or V299L *BCR::ABL* mutation, those with inflammatory bowel disease, those who received cytochrome P-450 CYP3A4 inhibitors or inducers, those infected with human immunodeficiency virus and/or hepatitis B/C virus, those who were pregnant or breastfeeding, and those with critical complications (as defined by the attending physician) were excluded. The trial was approved by the institutional review board of each participating hospital (UMIN 000032282). All procedures involving human participants were undertaken in accordance with the principles of the Declaration of Helsinki, and all participants provided written informed consent. The protocol is available in the appendix (Supplementary appendix).

### Procedures

After enrollment, patients received an initial daily dose of bosutinib (200 mg) orally. If no grade 3 or higher AEs occurred, the dose was escalated by 100 mg daily (up to 500 mg daily) every 2 weeks (Supplementary appendix). Major *BCR::ABL* mRNA levels were measured using a commercially available quantitative reverse transcription PCR (RT-qPCR) kit. Measurements were made at a central laboratory (SRL, Tokyo, Japan or Bio Medical Laboratories, Tokyo, Japan) and in accordance with the standardized international scale (IS). Plasma trough concentrations of bosutinib were measured at inVentive Health Clinical (NJ, USA) using a validated liquid chromatography/tandem mass spectrometry (LC/MS/MS) assay. Major *BCR::ABL* mRNA levels and plasma trough concentrations of bosutinib were measured every 3 months (i.e., at 3, 6, 9, and 12 months post-initiation). Two or three-color flow cytometry was performed (Bio Medical Laboratories, Tokyo, Japan) at 3, 6, and 12 months post-initiation to assess T cell and natural killer (NK) cell profiles. T cell and natural killer cell subsets were defined as follows: CD8 T cells = CD3^+^CD8^+^; NK cells = CD3^−^ CD56^+^ and CD16^+^ CD56^+^; and regulatory T (Treg) cells = CD4^+^ CD25^+^ CD127^−^.

The primary endpoint was the rate of treatment discontinuation at 12 months due to DRTs. Secondary endpoints included the treatment interruption rate, the mean bosutinib dose, number of dosing days and relative dose intensity up until 12 months, achievement of a cytogenetic response (CCyR; equivalent for *BCR::ABL1* mRNA ≦ 1.0%) at 6 and 12 months, and the cumulative rates of major molecular response (MMR; *BCR::ABL1* mRNA ≦ 0.1%) or deep molecular response (DMR; *BCR::ABL1* mRNA ≦ 0.01%) at 3, 6, 9 and 12 months. Sample size was calculated using research and biostatistics Cancer Research and Biostatistics SWOG statistical tools (null proportion = 0.32; alternative proportion = 0.14; one-sided α error = 5%; β error = 20%; power 80%), with the discontinuation rate of the previous Japanese phase 1/2 study of bosutinib as a reference [10]. Considering protocol deviations, the official target enrollment sample size for this trial was 35.

### Statistical analysis

For the primary endpoint, the rate of bosutinib discontinuation at 12 months due to DRTs and the one-sided 95% confidence interval (CI) were estimated using the Wilson method. The binomial test was used to evaluate whether the percentage was <32%. For the safety and secondary endpoints, Fisher’s exact test was used for categorical variables and the Mann–Whitney test for continuous variables. The Pearson correlation coefficient is used to determine correlation between bosutinib trough concentrations and several variables. Significant differences between the three groups were assessed using one-way ANOVA (post-hoc tests using Bonferroni), and those between two groups were assessed using the Mann–Whitney *U* test. The MMR and rate of DMR, as well as the two-sided 95% CI, were also estimated using the Wilson method. Receiver operating characteristic (ROC) curve analysis was done to determine optimal bosutinib concentration. The significance level for the primary hypothesis test was 0.05 (one-sided), and that for all other secondary endpoints was 0.05 (two-sided). Statistical analyses were performed using SAS version 9.4 (SAS Institute Inc., Cary, NC, USA). The study is registered with the UMIN Clinical Trials Registry (UMIN 000032282).

## Results

### Patient characteristics

Between February 4, 2019 and May 24, 2022, 35 patients from four Japanese hospitals were enrolled. The median age was 61 years (range, 26–81); 19 patients were male and 16 were female, and 16, 12, and seven had low, intermediate, and high Sokal scores, respectively. 11 patients were resistant to prior therapy and 24 were intolerant, respectively. The numbers of patients previously treated with TKIs were as follows: 19 (54%) received one TKI, 12 (34%) received three, and four (11%) received three. Prior TKIs were imatinib (*n* = 8, 23%), dasatinib (*n* = 27, 77%), nilotinib (*n* = 16, 46%), and bosutinib (*n* = 1, 3%, the patient who received prior treatment with bosutinib was intolerant to standard dosage of bosutinib); 23 of 35 patients (66%) had medical complications, which included hypertension (10, 29%), diabetes mellitus (five, 14%), cardiovascular disease (three, 9%), solid tumors (two, 6%), and cerebrovascular disease (one, 3%). The median major *BCR::ABL* mRNA level was 0.1% (0.0–34.6%), and serum aspartate aminotransferase (AST) and alanine aminotransferase (ALT) levels were 23 U/L (11–63) and 20 U/L (12–94), respectively (Table 1).

**Table 1:** Patient characteristics

	*n* (%), median (range)
Age (years)	61 (26–81)
Sex, male	19 (54)
Sokal risk	
Low	16 (46)
Intermediate	12 (34)
High	7 (20)
Numbers of prior TKIs	
One	19 (54)
Two	12 (34)
Three	4 (11)
Prior TKIs	
Imatinib	8 (23)
Dasatinib	27 (77)
Nilotinib	16 (46)
Bosutinib	1 (3)
*BCR::ABL1* mRNA %	0.1 (0–34.6)
100–10%	6 (17)
10–1.0%	5 (14)
1.0–0.1%	6 (17)
0.1–0.01%	10 (29)
0.01–0.0032%	4 (11)
0.0032–0%	4 (11)
AST (IU/L)	23 (11–63)
ALT (IU/L)	20 (12–94)
Complications	23 (66)
Hypertension	10 (29)
Diabates millitus	5 (14)
Cardiovascular disease	3 (9)
Solid tumors	2 (6)
Cerebrovascular disease	2 (6)
Liver disease	0 (0)
Autoimmune disorders	0 (0)
Others	15 (43)
Reason of switching to bosutinib	
Resistant to prior TKI	11 (31)
Intolerant to prior TKI	24 (69)

### The bosutinib discontinuation rate due to DRTs was lower than that reported previously

**Fig. 1:**
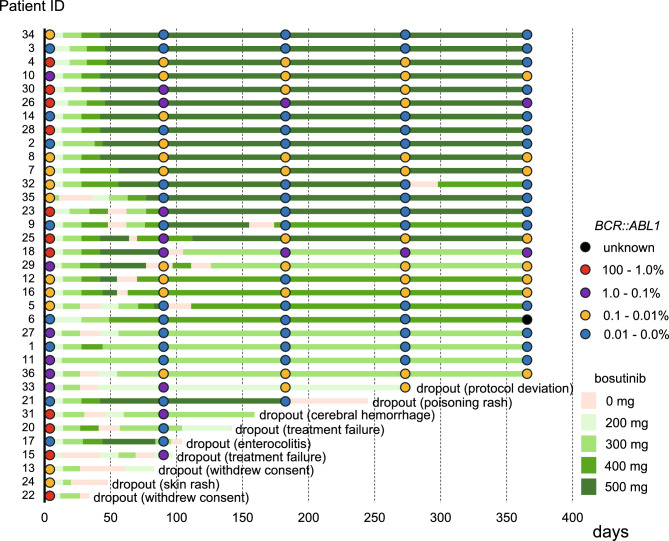
Swimmer plot displaying the treatment course. Each bar represents an individual patient

In the intention to treat population, nine of 35 patients had discontinued bosutinib at 12 months; four of these discontinuations were due to DRTs. The total bosutinib discontinuation rate was 25.7% ([95% CI, 15.6–39.3%] vs. 35.9% (14/39) for the Japanese phase 1/2 study [10]; *p* = 0.102), and the bosutinib discontinuation rate due to DRTs was 11.4% ([95% CI, 5.2–23.2%] vs. 28.2% (11/39, *p* = 0.015); the differences were significant. Common grade 3 to 4 AEs were consistent with the known safety profiles of bosutinib; these included skin rash (26%), increased AST (9%), and increased ALT (20%); there were no differences in grade 3–4 skin rash (*p* = 1.00), increased AST (*p* = 0.690), and increased ALT (*p* = 0.554) between the present study and the previous the Japanese phase 1/2 study; the incidence of diarrhea was low (3% vs. 25% in the Japanese phase 1/2 study; *p* = 0.018)). No pleural effusion (0%), cardiovascular events (0%), and only one cerebral hemorrhage (3%), were observed (Table 2). These results suggest that a lower initiating dose of bosutinib reduces the discontinuation rate due to DRTs; thus, the primary endpoint was met. A total of 21 patients (60.0%) interrupted bosutinib at least once due to grade 3 or higher AEs, except for one patient who discontinued due to a grade 2 pleural effusion at the decision of the physician. The other reasons for interruptions were skin rash (*n* = 9), liver dysfunction (*n* = 7), diarrhea (*n* = 1), appetite loss (*n* = 1), acute enterocolitis (*n* = 1), increased lipase (*n* = 1), nausea (*n* = 1), general fatigue (*n* = 1), cerebral hemorrhage (*n* = 1), poisoning rash (*n* = 1) and anemia (*n* = 1). Median bosutinib interruption duration was 16 days (range, 6–64). The mean and median bosutinib dose, the number of bosutinib dosing days, and the relative dose intensity up until 12 months were 388.7 (204.9–477.3) mg/day, 391.7 (204.9–477.3) mg, 351.0 (20–365) days, and 78% (41–95), respectively. A swimmer plot detailing treatment duration and outcomes for individuals in the study is shown in Fig. 1.

**Table 2:** Adverse events and laboratory abnormalities during bosutinib treatment (grades 3–4)

	*n*	%
Adverse events	20	57
Leukocytopenia	0	0
Lymphopenia	2	6
Neutropenia	1	3
Anemia	1	3
Thrombocytopenia	0	0
Skin rash	9	26
Nausea, vomiting	1	3
Diarrhea	1	3
AST increased	3	9
ALT increased	7	20
Increased lipase	1	3
Acute enterocolitis	1	3
Cerebral hemorrhage	1	3
Appetite loss	1	3
Fatigue	1	3
Poisoning rash	1	3

### Bosutinib efficacy was high even at the lower initiating dose

**Fig. 2:**
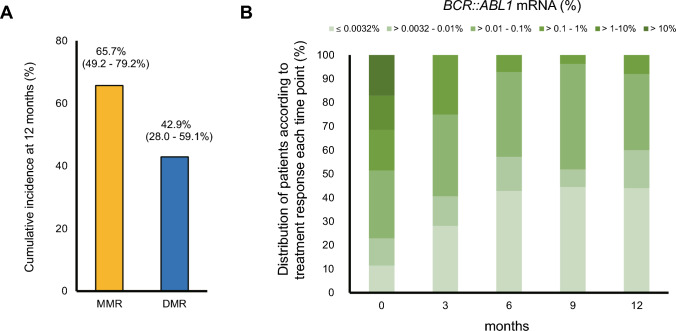
**A** Major molecular response (MMR) and deep molecular response (DMR) at 12 months. The graph shows the MMR or DMR rates at 12 months after initiation of bosutinib treatment. **B** Dose distribution for all 35 patients from baseline until 12 months

*BCR::ABL1* mRNA ≦1% was achieved by 28/35 patients (80.0%) at 6 months, and by 26/35 patients (74.3%) at 12 months. Notably, all patients who could continue bosutinib until 12 months achieved *BCR::ABL1* mRNA ≦1%, equivalent to a CCyR. The cumulative incidences of MMR and DMR at 12 months were 23/35 (65.7%; 95% CI, 49.2–79.2%) and 15/35 (42.9%; 95% CI, 28.0–59.1%), respectively (Fig. 2A). For those who could be evaluated. The cumulative incidence of a molecular response at each time point is presented in Fig. 2B. Taken together, the results suggest that bosutinib efficacy remains high in CML-CP patients who were resistant and/or intolerant to prior therapy, even when used at a lower initiating dose.

### PK of bosutinib

**Fig. 3:**
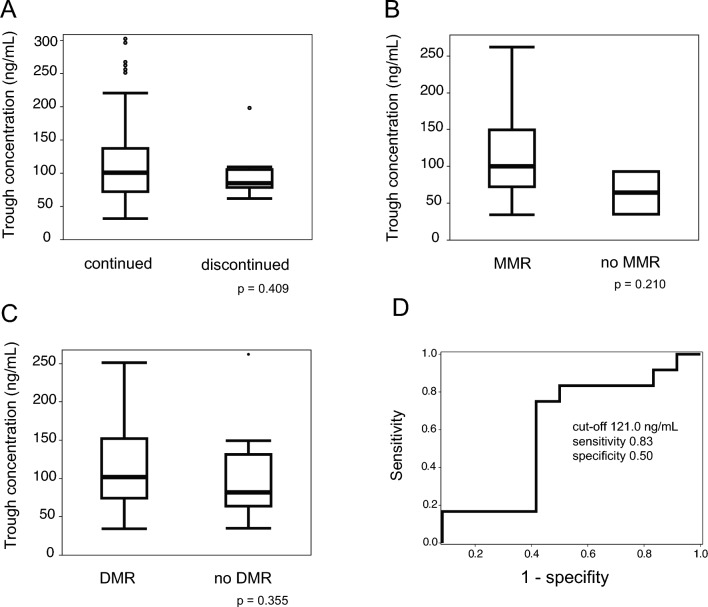
Pharmacokinetics of bosutinib. Trough concentrations of bosutinib in patients who continued treatment (**A**), or achieved of major molecular response (MMR, **B**) or a deep molecular response (DMR, **C**). ROC curve shows that the optimal trough concentration required for a 1 log reduction was 121.0 ng/mL (**D**). IS, international scale

The average trough concentration of bosutinib at 3, 6 and 12 months was 114.4, 118.0 and 112.6 ng/mL, respectively. When we investigated factors that can affect the bosutinib trough concentration (i.e., patient age, sex, body weight, body mass index, liver function, and renal function), we found that none did (Table S1). Furthermore, there was no difference in average trough concentrations between those who continued (26 patients) and those that discontinued treatment due to AEs (six patients): 116.9 vs. 98.9 ng/mL, respectively (*p* = 0.409, Mann–Whitney *U* test; Fig. 3A). With respect to an association between achievement of MMR or DMR and the bosutinib trough concentration, we found that patients who achieved molecular remission tended to have higher trough concentrations: MMR = 117.0 vs. 64.1 ng/mL, respectively (*p* = 0.210; Fig. 3B) and DMR = 117.7 vs. 104.0 ng/mL, respectively (*p* = 0.355; Fig. 3C). The optimal trough concentration for a 1 log reduction was 121.0 ng/mL (based on ROC curve analysis and Youden index criteria) (Fig. 3D).

### T cell and NK cell dynamics after initiation of bosutinib treatment

**Fig. 4:**
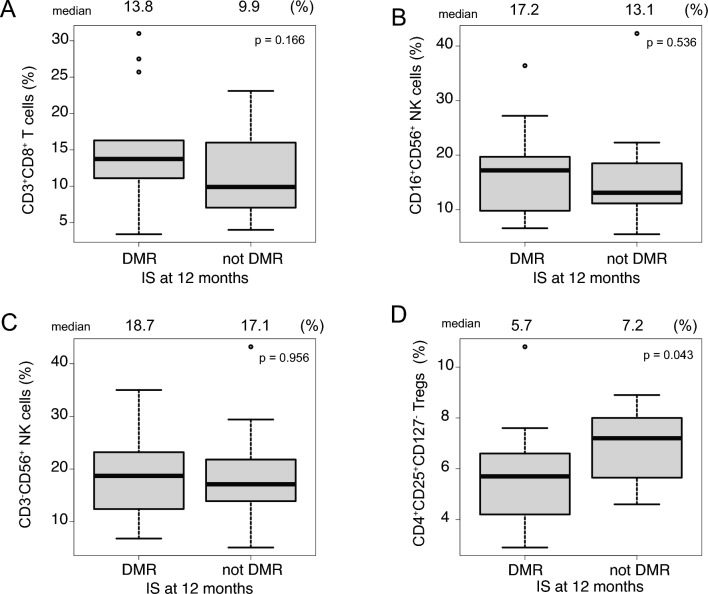
Flow cytometry analysis of T cells and natural killer (NK) cells at 12 months post-treatment initiation. Percentage of CD3^+^ T cells (**A**), CD16^+^CD56^+^ NK cells (**B**), CD3^−^CD56^+^NK cells (**C**), and regulatory T cells according to the molecular response (**D**). DMR, deep molecular response

We performed flow cytometry for sequential assessment of changes in the T cell and NK cell profiles following initiation of bosutinib treatment. The percentages of CD8^+^ T cells, CD3^−^CD56^+^ NK cells, CD16^+^CD56^+^ NK cells, and Tregs changed little after bosutinib initiation (Figure S1). Next, we evaluated the association between immune cell numbers and bosutinib treatment outcomes. There were no differences in the percentage of CD8+ T cells and NK cells between patients who achieved DMR and those that did not (Fig. 4A–C); however, patients who achieved DMR at 12 months had a lower percentage of Tregs than those who did not (5.7% vs. 7.2%, respectively; *p* = 0.043; Fig. 4D). These results indicate that bosutinib does not affect most immune cell populations, whereas the low numbers of Tregs might play a role in patient responses to bosutinib.

## Discussion

International prospective clinical trials show that bosutinib is a safe and effective second-generation TKI for patients with newly diagnosed or pre-treatment refractory-intolerant CML-CP [4–7], although DRT forces dose reduction in some patients; however, sub-analyses indicate an adequate molecular response in such patients [12, 13]. Establishing the optimal dose of bosutinib is necessary to achieve optimal efficacy while avoiding AEs; therefore, some experts recommend a lower initiating dose [8].

Mita et al. [14] conducted a retrospective study comparing a dose-escalation regimen of bosutinib versus a standard dose starting regimen. The initiating dose of bosutinib was 100 mg daily, escalating to 500 mg daily; the result was a reduction in interruption or discontinuation rates due to AEs when compared with the standard 500 mg daily fixed dose [14]. The recent BODO study, a phase 2 trial examining the safety and efficacy of step-in dosing of bosutinib [9], reported that a 300 mg daily initiation regimen failed to reduce gastrointestinal AEs or discontinuation rates; 17 of the 57 patients (29.8%) discontinued bosutinib due to AEs [9]. In the present BOGI trial, the initial dose of bosutinib was 200 mg daily; subsequently, the dose was escalated by 100 mg daily (up to 500 mg daily) every 2 weeks. The primary endpoint was the bosutinib discontinuation rate due to DRTs at 12 months; the result was 11.4%; hence, the primary endpoint was met. The lower rate in the BOGI trial compared with that in the Japanese phase 1/2 study [10] was attributed to an apparent reduction in the incidence of diarrhea. Furthermore, a previous study reported a correlation between the average bosutinib concentration during the early treatment phase (< 1 week) and occurrence of grade 3 diarrhea, suggesting that a lower initiating dose of bosutinib reduce diarrhea and therefore AEs [15]. These results suggest that a 300 mg daily initiating dose may be too high for a certain subset of patients, whereas 100 to 200 mg daily may be optimal [14, 16].

Approximately 10–20% of patients who receive bosutinib developed a grade 3 to 4 increase in transaminase levels, leading to discontinuation [4, 6, 7]; grade 3 to 4 increases in transaminase are more common in Japanese patients than in non-Japanese patients (36.2 vs. 17.7%, respectively); this leads directly to bosutinib discontinuation in 30.4% of Japanese patients [17]. In the BOGI trial, we noted a similar frequency of grade 3 to 4 increases in transaminase (AST, 9%; ALT, 20%) [4, 6, 7], although these did not lead to bosutinib discontinuation. Since it is not uncommon for initial elevations in transaminase to resolve spontaneously, close monitoring and early evaluation of liver enzymes, along with appropriate modifications in the drug dose, might have helped these patients to continue bosutinib throughout the BOGI trial. Furthermore, the median dose of bosutinib in patients enrolled in the BOGI trial was relatively higher (391.7 mg/day) than that in the previous Japanese clinical study (353.9 mg/day) [17], although the cumulative incidences of MMR and DMR at 12 months (MMR, 65.7%; DMR, 42.9%) were comparable with those in the BELA or BYOND trials [4, 7]; therefore, a lower initiating dose (followed by subsequent dose escalation) regimen can maintain dose intensity, resulting in an adequate therapeutic effect. Since the continuation rates and efficacy of bosutinib are associated [4, 6, 7, 18], frequent monitoring of liver enzyme along with dose modification is important to avoid bosutinib discontinuation due to increased transaminase levels while at the same time enabling optimal efficacy. Although the observation period was short, the low incidence of cardiovascular AEs in the BOGI trial was notable.

We also measured trough concentrations of bosutinib over time to identify the effective therapeutic range that exhibits optimal safety and efficacy. Unfortunately, we found no significant difference in the trough concentrations between patients who discontinued bosutinib due to AEs (*n* = 6) and those who were able to continue (*n* = 26). This may be due to the fact that few patients had their concentrations measured at the time of AE onset and/or at the time of bosutinib discontinuation. Only six of nine patients who discontinued bosutinib due to AEs had bosutinib concentrations measured at the time determined by the protocol. Bosutinib trough concentrations are associated with gastrointestinal toxicity; however, the target trough concentration needed to avoid AEs remains unclear [15]. Patients who achieved an MMR tended to have higher trough concentrations, a finding consistent with a previous report; the optimal trough concentration for a 1 log reduction in the BOGI trial was 121.0 ng/mL. Further PK studies are needed to establish target concentrations and the optimal individual doses require to ensure safety and efficacy.

Several lines of evidence suggest that CML is sensitive to immunotherapy [19], and that responses to TKI therapy can be determined not only by direct binding capacity for BCR::ABL but also by anti-tumor immune responses [20, 21]. Imatinib or dasatinib often increase NK cell numbers or decrease Treg numbers, leading to favorable clinical responses [22–24], while nilotinib or bosutinib affect immune cells only weakly [23]. Tregs, which are major mediators of mammalian self-tolerance, suppress anti-tumor immunity, making them obstacles to successful cancer treatment [25]. In the present study, we found that bosutinib did not affect T and NK cells, as reported previously [23, 26]; however, we found that the outcome of bosutinib treatment might be dependent on the number of Tregs. Lower Treg numbers are associated with successful achievement of a DMR [24, 27], as well as maintenance of a treatment free remission in CML patients because they activate anti-CML immune responses [28]. These results suggest that although bosutinib itself might have no direct effect on immune cells, immune cells may affect the therapeutic efficacy of bosutinib.

## Conclusion

A lower initiating dose of bosutinib reduces drug discontinuation rates, or interruption of treatment, due to severe DRTs, particularly diarrhea, while maintaining therapeutic efficacy. Although bosutinib concentration levels might correlate with safety and efficacy, the target trough concentrations required for optimal safety and efficacy remain unclear. Further large-scale studies are needed to further investigate the PK of bosutinib.

## Supplementary Information

Below is the link to the electronic supplementary material.

Supplementary file1 (DOCX 17 KB)

Supplementary file2 (PDF 209 KB)

Supplementary file3 (DOCX 165 KB)

## Data Availability

All data can be assessed by contacting the corresponding author (NT).

## References

[1] Hochhaus A, Larson RA, Guilhot F, Radich JP, Branford S, Hughes TP, et al. Long-term outcomes of imatinib treatment for chronic myeloid leukemia. N Engl J Med. 2017;376:917–27. 28273028 10.1056/NEJMoa1609324 PMC5901965

[2] Cortes JE, Saglio G, Kantarjian HM, Baccarani M, Mayer J, Boqué C, et al. Final 5-year study results of DASISION: the dasatinib versus imatinib study in treatment-Naïve chronic myeloid leukemia patients trial. J Clin Oncol. 2016;34:2333–40. 27217448 10.1200/JCO.2015.64.8899 PMC5118045

[3] Hochhaus A, Saglio G, Hughes TP, Larson RA, Kim DW, Issaragrisil S, et al. Long-term benefits and risks of frontline nilotinib vs. imatinib for chronic myeloid leukemia in chronic phase: 5-year update of the randomized ENESTnd trial. Leukemia. 2016;30:1044–54. 26837842 10.1038/leu.2016.5 PMC4858585

[4] Cortes JE, Kim DW, Kantarjian HM, Brümmendorf TH, Dyagil I, Griskevicius L, et al. Bosutinib versus imatinib in newly diagnosed chronic-phase chronic myeloid leukemia: results from the BELA trial. J Clin Oncol. 2012;30:3486–92. 22949154 10.1200/JCO.2011.38.7522 PMC4979199

[5] Cortes JE, Kantarjian HM, Brümmendorf TH, Kim DW, Turkina AG, Shen ZX, et al. Safety and efficacy of bosutinib (SKI-606) in chronic phase Philadelphia chromosome-positive chronic myeloid leukemia patients with resistance or intolerance to imatinib. Blood. 2011;118:4567–76. 21865346 10.1182/blood-2011-05-355594 PMC4916618

[6] Cortes JE, Gambacorti-Passerini C, Deininger MW, Mauro MJ, Chuah C, Kim DW, et al. Bosutinib versus imatinib for newly diagnosed chronic myeloid leukemia: results from the randomized BFORE trial. J Clin Oncol. 2018;36:231–7. 29091516 10.1200/JCO.2017.74.7162 PMC5966023

[7] Hochhaus A, Gambacorti-Passerini C, Abboud C, Gjertsen BT, Brümmendorf TH, Smith BD, et al. Bosutinib for pretreated patients with chronic phase chronic myeloid leukemia: primary results of the phase 4 BYOND study. Leukemia. 2020;34:2125–37. 32572189 10.1038/s41375-020-0915-9 PMC7387243

[8] Cortes JE, Apperley JF, Deangelo DJ, Deininger MW, Kota VK, Rousselot P, et al. Management of adverse events associated with bosutinib treatment of chronic-phase chronic myeloid leukemia: expert panel review. J Hematol Oncol. 2018;11:1–12. 30587215 10.1186/s13045-018-0685-2 PMC6307238

[9] Isfort S, Manz K, Teichmann LL, Crysandt M, Burchert A, Hochhaus A, et al. Step-in dosing of bosutinib in pts with chronic phase chronic myeloid leukemia (CML) after second-generation tyrosine kinase inhibitor (TKI) therapy: results of the Bosutinib Dose Optimization (BODO) Study. Ann Hematol. 2023;102:2741–52. 37592092 10.1007/s00277-023-05394-0 PMC10492675

[10] Nakaseko C, Takahashi N, Ishizawa K, Kobayashi Y, Ohashi K, Nakagawa Y, et al. A phase 1/2 study of bosutinib in Japanese adults with Philadelphia chromosome-positive chronic myeloid leukemia. Int J Hematol. 2015;101:154–64. 25540064 10.1007/s12185-014-1722-8

[11] Abbas R, Hug BA, Leister C, El GM, Chalon S, Sonnichsen D. A phase I ascending single-dose study of the safety, tolerability, and pharmacokinetics of bosutinib (SKI-606) in healthy adult subjects. Cancer Chemother Pharmacol. 2012;69:221–7. 21691746 10.1007/s00280-011-1688-7

[12] Brummendorf TH, Gambacorti-Passerini C, Hochhaus A, Lipton JH, Kota V, Deininger MW, et al. Efficacy and safety following dose reduction of bosutinib or imatinib in patients with newly diagnosed chronic myeloid leukemia: analysis of the phase 3 BFORE trial. Blood. 2018;132:3005.

[13] Kota V, Brümmendorf TH, Gambacorti-Passerini C, Lipton JH, Kim D-W, An F, et al. Efficacy and safety following bosutinib dose reduction in patients with Philadelphia chromosome-positive leukemias. Leuk Res. 2021;111: 106690. 34673442 10.1016/j.leukres.2021.106690

[14] Mita A, Abumiya M, Miura M, Niioka T, Takahashi S, Yoshioka T, et al. Correlation of plasma concentration and adverse effects of bosutinib: standard dose or dose-escalation regimens of bosutinib treatment for patients with chronic myeloid leukemia. Exp Hematol Oncol. 2018;7:9. 29682402 10.1186/s40164-018-0101-1 PMC5899348

[15] Garrett M, Knight B, Cortes JE, Deininger MW. Population modeling of bosutinib exposure-response in patients with newly diagnosed chronic phase chronic myeloid leukemia. Cancer Med. 2023;12:17981–92. 37553873 10.1002/cam4.6439 PMC10524044

[16] Kantarjian HM, Jabbour EJ, Lipton JH, Castagnetti F, Brümmendorf TH. A review of the therapeutic role of bosutinib in chronic myeloid leukemia. Clin Lymphoma Myeloma Leuk. 2024;24:285–97. 38278737 10.1016/j.clml.2024.01.005

[17] Takahashi N, Cortes JE, Sakaida E, Ishizawa K, Ono T, Doki N, et al. Safety profile of bosutinib in Japanese versus non-Japanese patients with chronic myeloid leukemia: a pooled analysis. Int J Hematol. 2022;115:838–51. 35235189 10.1007/s12185-022-03314-y

[18] Hochhaus A, Réa D, Boquimpani C, Minami Y, Cortes JE, Hughes TP, et al. Asciminib vs. bosutinib in chronic-phase chronic myeloid leukemia previously treated with at least two tyrosine kinase inhibitors: longer-term follow-up of ASCEMBL. Leukemia. 2023;37:617–26. 36717654 10.1038/s41375-023-01829-9 PMC9991909

[19] Müller MC, Gattermann N, Lahaye T, Deininger MWN, Berndt A, Fruehauf S, et al. Dynamics of BCR-ABL mRNA expression in first-line therapy of chronic myelogenous leukemia patients with imatinib or interferon alpha/ara-C. Leukemia. 2003;17:2392–400. 14523462 10.1038/sj.leu.2403157

[20] Hughes A, Clarson J, Tang C, Vidovic L, White DL, Hughes TP, et al. CML patients with deep molecular responses to TKI have restored immune effectors and decreased PD-1 and immune suppressors. Blood. 2017;129:1166–76. 28049640 10.1182/blood-2016-10-745992

[21] Ureshino H, Shindo T, Kimura S. Role of cancer immunology in chronic myelogenous leukemia. Leuk Res. 2020;88:106273. 31765938 10.1016/j.leukres.2019.106273

[22] Fei F, Yu Y, Schmitt A, Rojewski MT, Chen B, Götz M, et al. Dasatinib inhibits the proliferation and function of CD4^+^ CD25^+^ regulatory T cells. Br J Haematol. 2009;144:195–205. 19016717 10.1111/j.1365-2141.2008.07433.x

[23] Kreutzman A, Yadav B, Brummendorf TH, Gjertsen BT, Lee Hee M, Janssen J, et al. Immunological monitoring of newly diagnosed CML patients treated with bosutinib or imatinib first-line. Oncoimmunology. 2019;8:1–13. 10.1080/2162402X.2019.1638210 PMC6685516 31428530

[24] Najima Y, Yoshida C, Iriyama N, Fujisawa S, Wakita H, Chiba S, et al. Regulatory T cell inhibition by dasatinib is associated with natural killer cell differentiation and a favorable molecular response—the final results of the D-first study. Leuk Res. 2018;66:66–72. 29407585 10.1016/j.leukres.2018.01.010

[25] Tay C, Tanaka A, Sakaguchi S. Tumor-infiltrating regulatory T cells as targets of cancer immunotherapy. Cancer Cell. 2023;41:450–65. 36917950 10.1016/j.ccell.2023.02.014

[26] Marinelli Busilacchi E, Costantini A, Viola N, Costantini B, Olivieri J, Butini L, et al. Immunomodulatory effects of tyrosine kinase inhibitor in vitro and in vivo study. Biol Blood Marrow Transplant. 2018;24:267–75. 29128554 10.1016/j.bbmt.2017.10.039

[27] Tanaka A, Nishikawa H, Noguchi S, Sugiyama D, Morikawa H, Takeuchi Y, et al. Tyrosine kinase inhibitor imatinib augments tumor immunity by depleting effector regulatory T cells. J Exp Med. 2020;217:1–14. 31704808 10.1084/jem.20191009 PMC7041710

[28] Okada M, Imagawa J, Tanaka H, Nakamae H, Hino M, Murai K, et al. Final 3-year results of the dasatinib discontinuation trial in patients with chronic myeloid leukemia who received dasatinib as a second-line treatment. Clin Lymphoma, Myeloma Leuk. 2018;18:353–360.e1. 29610029 10.1016/j.clml.2018.03.004

