# Supplementary material for: A lower initial dose of bosutinib for patients with chronic myeloid leukemia patients resistant and/or intolerant to prior therapy: a single-arm, multicenter, phase 2 trial (BOGI trial)
Source: Int J Hematol. 2024 Aug 13;120(4):492–500. doi: 10.1007/s12185-024-03830-z (PMC11415413; doi:10.1007/s12185-024-03830-z)
Supplement: Supplementary file 1 — Supplementary file1 (DOCX 17 KB) [file 12185_2024_3830_MOESM1_ESM.docx]

**Table S1. Correlation analysis between bosutinib trough concentrations and several variables**

| Variable | n | Correlation coefficient r | Lower limit 95% | Upper limit 95% | p value |
| --- | --- | --- | --- | --- | --- |
| Age | 24 | 0.15 | -0.27 | 0.52 | 0.494 |
| Sex | 24 | 0.04 | -0.37 | 0.43 | 0.863 |
| Body weight | 24 | 0.11 | -0.31 | 0.49 | 0.617 |
| Body mass index | 24 | 0.09 | -0.32 | 0.48 | 0.670 |
| Creatinine (mg/dL) | 24 | 0.04 | -0.37 | 0.44 | 0.843 |
| AST (U/L) | 24 | 0.15 | -0.27 | 0.52 | 0.495 |
| ALT (U/L) | 24 | 0.04 | -0.37 | 0.44 | 0.841 |
