# Supplementary figures and images for: A lower initial dose of bosutinib for patients with chronic myeloid leukemia patients resistant and/or intolerant to prior therapy: a single-arm, multicenter, phase 2 trial (BOGI trial)
Source: Int J Hematol. 2024 Aug 13;120(4):492–500. doi: 10.1007/s12185-024-03830-z (PMC11415413; doi:10.1007/s12185-024-03830-z)

Figure S1

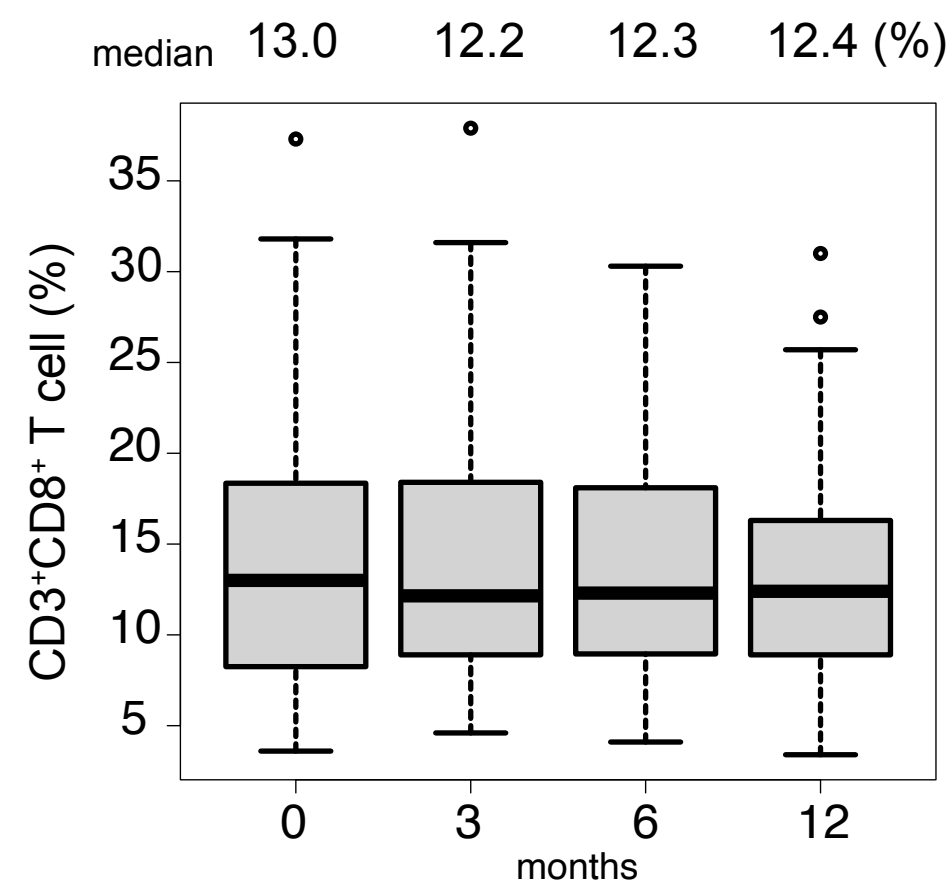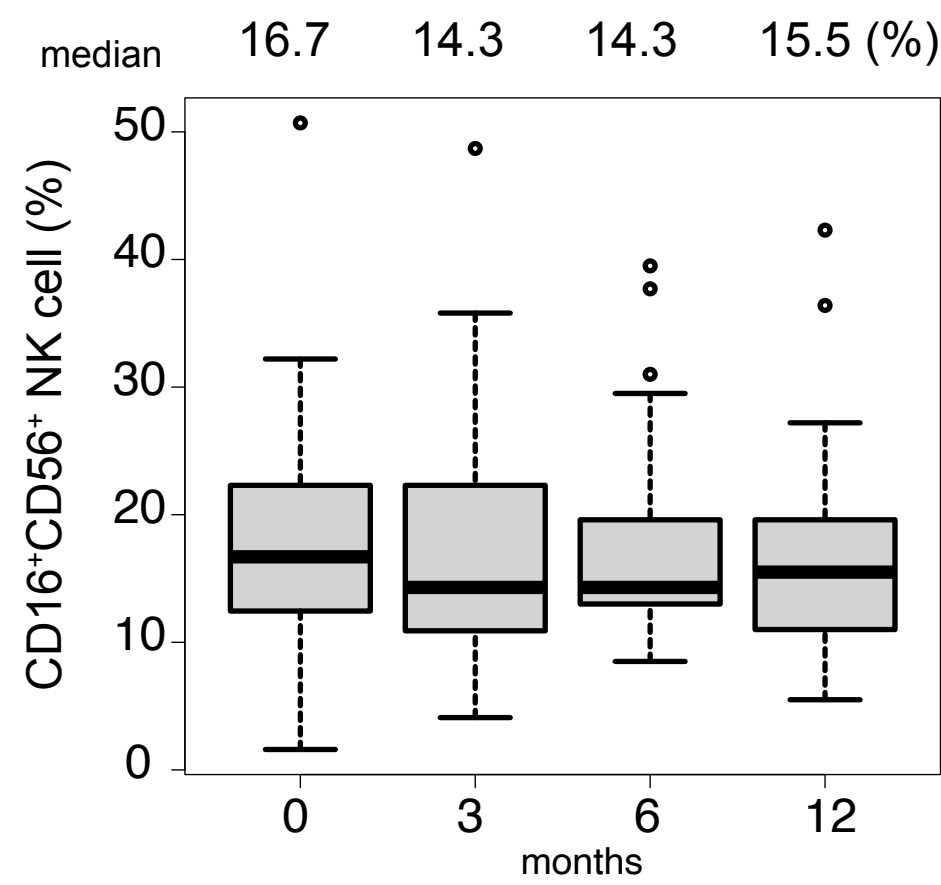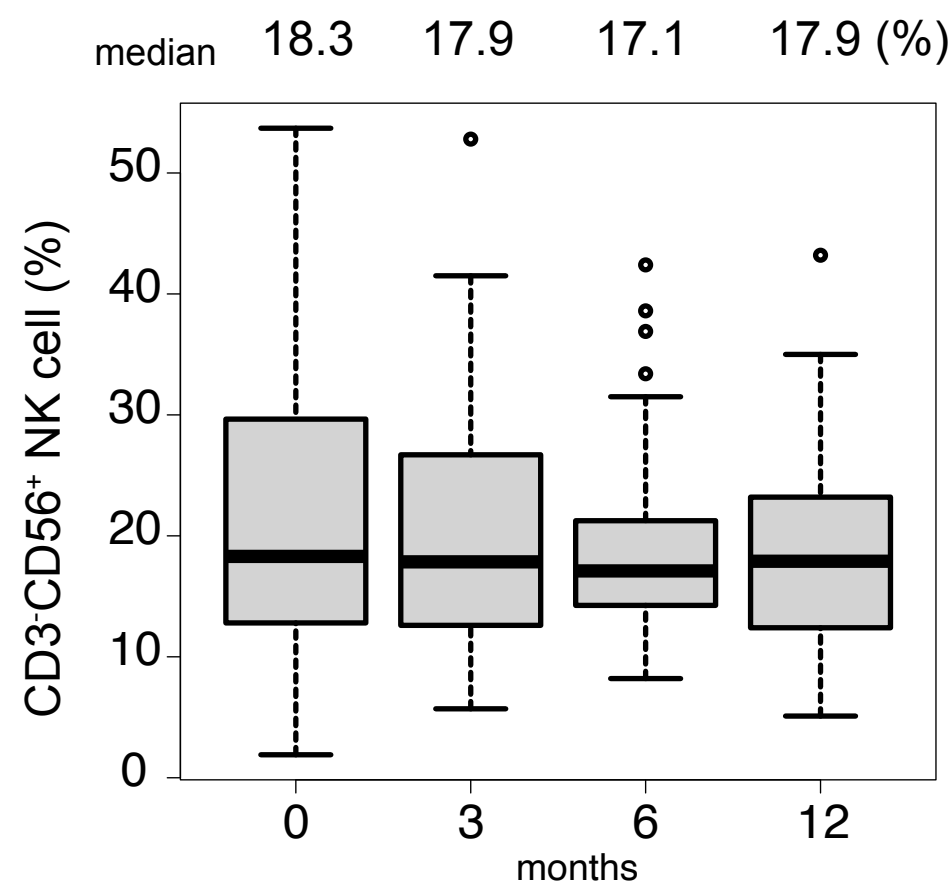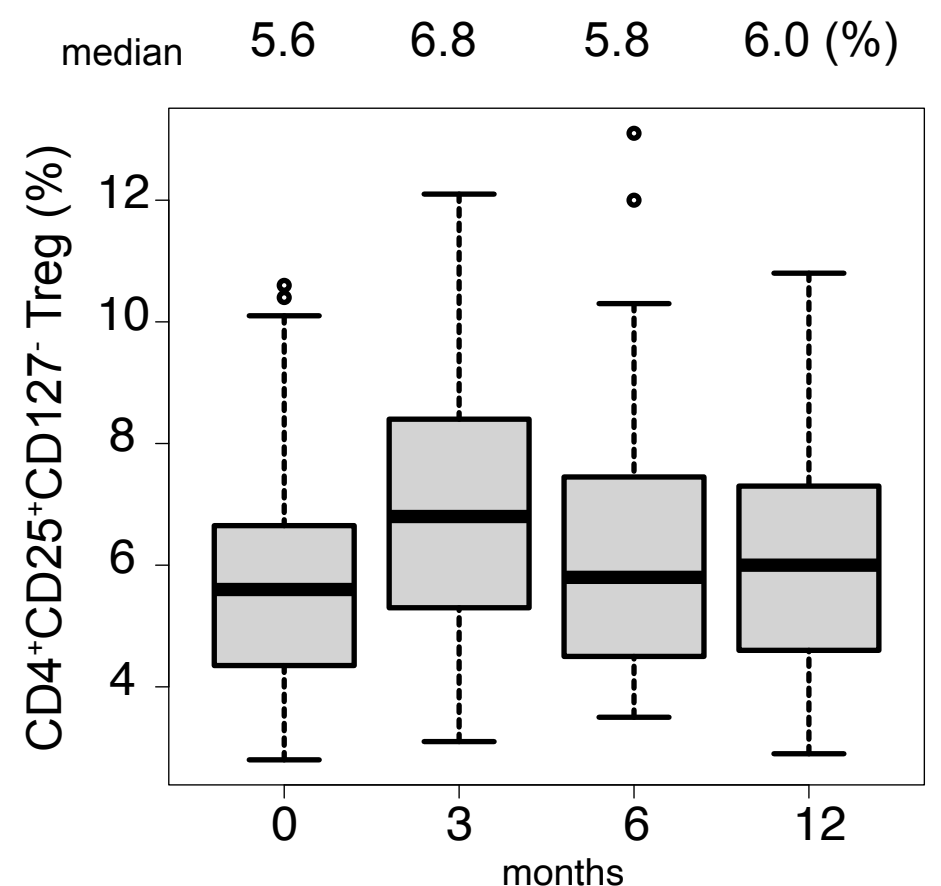

Figure S1. T cell and natural killer cell dynamics after bosutinib initiation.

Supplement: Supplementary file 2 — Supplementary file2 (PDF 209 KB) [file 12185_2024_3830_MOESM2_ESM.pdf]
