# Supplementary material for: A lower initial dose of bosutinib for patients with chronic myeloid leukemia patients resistant and/or intolerant to prior therapy: a single-arm, multicenter, phase 2 trial (BOGI trial)
Source: Int J Hematol. 2024 Aug 13;120(4):492–500. doi: 10.1007/s12185-024-03830-z (PMC11415413; doi:10.1007/s12185-024-03830-z)
Supplement: Supplementary file 3 — Supplementary file3 (DOCX 165 KB) [file 12185_2024_3830_MOESM3_ESM.docx]

**A phase 2 study of BOsutinib Gradual Increase as a second or third line treatment for chronic myeloid leukemia in chronic phase (BOGI Trial)**

**Protocol**

Co-Principal Investigator/owner：Shinya Kimura, MD, PhD.

Division of Hematology, Respiratory Medicine and Oncology,

Department of Internal Medicine, Faculty of Medicine, Saga University

5-1-1 Nabeshima, 849-8501 Saga, Japan

TEL: 0952-34-2366, FAX: 0952-34-2017

E-mail: shkimu@cc.saga-u.ac.jp

Co-Principal Investigator：Naoto Takahashi, MD, PhD.

Department of Hematology, Nephrology, and Rheumatology,

Akita University Graduate School of Medicine

1-1-1 Hondo, 010-8543 Akita, Japan

TEL: 018-884-6111, FAX: 018-836-2613

E-mail: naotot@doc.med.akita-u.ac.jp

Research Administration Bureau：Shinya Kimura, MD, PhD.

Division of Hematology, Respiratory Medicine and Oncology,

Department of Internal Medicine, Faculty of Medicine, Saga University

5-1-1 Nabeshima, 849-8501 Saga, Japan

TEL: 0952-34-2366, FAX: 0952-34-2017

E-mail: shkimu@cc.saga-u.ac.jp

# 0. Overview

## **0.1. Schema**


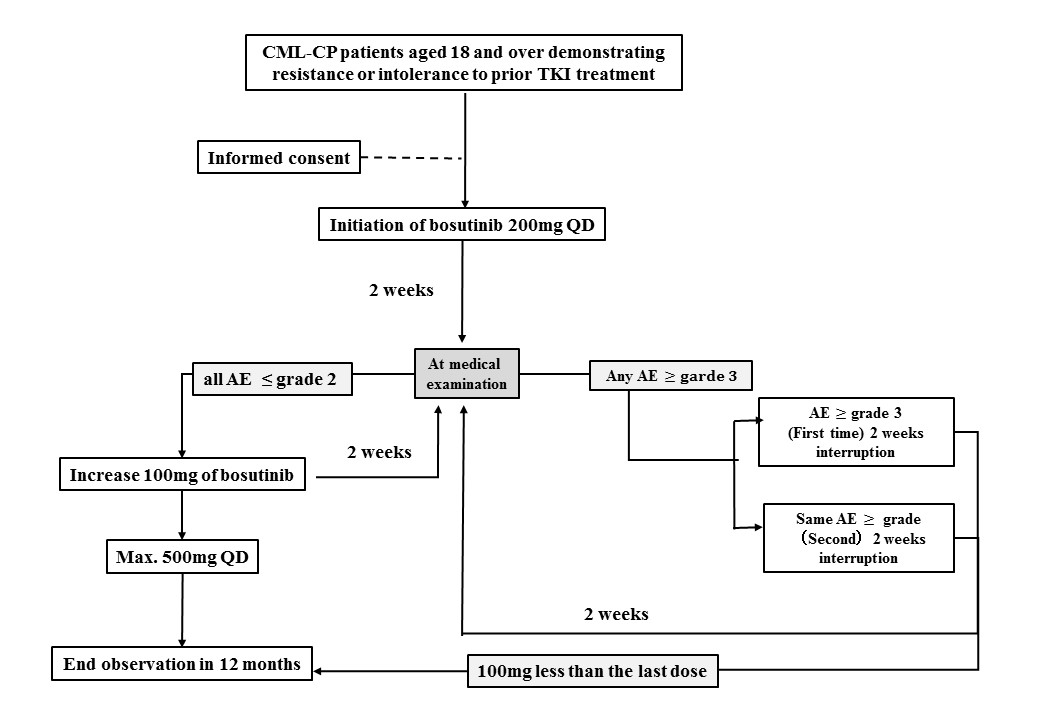


## **0.2. Purpose**

When the standard dose of bosutinib of 500mg QD is used as second or third line treatment for chronic myeloid leukemia in chronic phase (CML-CP) patients who have showed resistance or intolerance to prior ABL tyrosine kinase inhibitors (TKIs), severe diarrhea or liver failure occurs highly frequently, often requiring a dose interruption or discontinuation of treatment. We herein investigate whether gradual increase of bosutinib after starting at a low dose (200mg QD) can lower the dose interruption or discontinuation of treatment.

## **0.3. Endpoint**

### 0.3.1. Primary endpoint:

Bosutinib treatment drop-out rate due to AEs by 12 months after initiation of bosutinib

### 0.3.2. Secondary endpoints:

1. Rate of treatment interruptions
2. Mean bosutinib doses at 12 months after initiation of bosutinib
3. Administration period of bosutinib and its median dose intensity/relative dose intensity up to 12 months after initiation of bosutinib
4. Cumulative complete cytogenetic response (CCyR) maintenance rate at 6 and 12 months after initiation of bosutinib
5. Cumulative major molecular response (MMR) rate and cumulative deep molecular response (DMR rate) at 3, 6, 9 and 12 months after initiation of bosutinib
6. Incidence of all grades or grade 3 or 4 adverse events (AE)

### 0.3.3. Exploratory endpoint

1. Peripheral blood lymphocyte profiling from bosutinib administration
2. Trough concentration levels of bosutinib, AEs and molecular responses

## **0.4. Subjects**

CML-CP patients aged 18 and over demonstrating resistance or intolerance to prior TKI treatment

## **0.5. Treatment**

Oral administration of bosutinib is started at an initial dose of 200mg QD. If all AE are ≤ grade 2, the dose is gradually increased by 100mg/day every two weeks. If any of the AEs are ≥ grade 3, the administration is interrupted until the relevant AE returns to ≤ grade 1, and once the AE which caused the titration, becomes ≤ grade 1, the administration is restarted from the decreased dose (100mg less than the last dose) (e.g.: if a grade 3 AE occurs at 300mg QD, the administration is interrupted until the AE lowers to ≤ grade 1, and restarted from 200mg QD). If any of the AEs are ≥ grade 3 at the initial dose of 200mg, the administration will restart at the same dose (200mg QD) when the AE lowers to ≤ grade 1. While all AEs are maintained at ≤ grade 2 again, the dose is titrated again by 100mg every two weeks. This method of titration is continued until the daily dose reaches 500mg. When the same ≥ grade 3 AE is observed for two consecutive administrations of bosutinib at the same dose, a decreased dose (100mg less than that dose) is given as the maintenance dose. If the subject cannot take ≥ 300mg QD 3 months after initiation of bosutinib, then this protocol is terminated and it is switched to method of treatment other than bosutinib. Furthermore, the protocol is terminated if a ≥ grade 3 AE cannot be improved to ≤ grade 1 for more than four weeks despite interruption, and it is switched to the method of treatment other than bosutinib.

If it is judged as fail based on European LeukemiaNet (ELN) diagnosis criteria at 3, 6 and 12 months after initiation of bosutinib (efficacy criteria of TKI treatment for the second line treatment on patients for whom imatinib treatment for **CML (ELN 2013 version;** Appendix Table 1), this protocol is interrupted and the treatment is switched to something other than bosutinib. Subjects do not take bosutinib on the day of the visit and after checking for any adverse events by blood tests and clinical observations, the subject restarts treatment based on principal physician’s instructions at the dose prescribed by the protocol.

## **0.6. Scheduled enrolment and length of study**

Scheduled enrolled number：35 formally enrolled cases

Enrollment period： One year from the date of ethics committee approval

Follow-up period：12 months

Total length of study： 24 months

## **0.7. Contacts**

For inquiries on the test such as patient selection criteria:：

Research Administration Bureau：Shinya Kimura, MD, PhD.

Division of Hematology, Respiratory Medicine and Oncology,

Department of Internal Medicine, Faculty of Medicine, Saga University

5-1-1 Nabeshima, 849-8501 Saga, Japan

TEL: 0952-34-2366, FAX: 0952-34-2017

E-mail: shkimu@cc.saga-u.ac.jp

Data center: Hisako Yoshida

Clinical Research Center, Saga University Hospital

5-1-1, Nabeshima, 849-8501 Saga, Japan

TEL: 0952-34-3400, FAX: 0952-34-2085

E-mail: hyoshida@cc.saga-u.ac.jp

**Table of Contents**

[0. Overview 2](#_Toc489437263)

[**0.1. Schema** 2](#_Toc489437264)

[**0.2. Purpose** 2](#_Toc489437265)

[**0.3. Endpoint** 2](#_Toc489437266)

[0.3.1. Primary endpoint: 2](#_Toc489437267)

[0.3.2. Secondary endpoints: 2](#_Toc489437268)

[0.3.3. Exploratory endpoint 3](#_Toc489437269)

[**0.4. Subjects** 3](#_Toc489437270)

[**0.5. Treatment** 3](#_Toc489437271)

[**0.6. Scheduled enrolment and length of study** 4](#_Toc489437272)

[**0.7. Contacts** 4](#_Toc489437273)

[1. Purpose and Endpoint 9](#_Toc489437274)

[**1.1. Purpose** 9](#_Toc489437275)

[**1.2. Endpoint** 9](#_Toc489437276)

[1.2.1. Primary Endpoint: 9](#_Toc489437277)

[1.2.2. Secondary endpoints: 9](#_Toc489437278)

[1.2.3. Exploratory endpoints: 9](#_Toc489437279)

[2. Background 9](#_Toc489437280)

[**2.1. Subjects** 9](#_Toc489437281)

[2.1.1. Target disease 9](#_Toc489437282)

[2.1.2. Basis for setting the population 10](#_Toc489437283)

[**2.2. Standard treatment** 11](#_Toc489437284)

[**2.3. Drugs used** 11](#_Toc489437285)

[**2.4. Treatment regimen of the trial** 11](#_Toc489437286)

[**2.5. Trial design** 12](#_Toc489437287)

[2.5.1. Basis for setting endpoint 12](#_Toc489437288)

[2.5.2. Trial design 13](#_Toc489437289)

[2.5.3. Clinical hypothesis and basis for setting enrolled sample 13](#_Toc489437290)

[**2.6. Summary of expected benefits and risks (disadvantages) from participating in the trial** 13](#_Toc489437291)

[2.6.1. Expected benefits 13](#_Toc489437292)

[2.6.2. Expected risks and disadvantages 14](#_Toc489437293)

[**2.7. Significance of this trial** 14](#_Toc489437294)

[3. Criteria and definitions used in the present trial 14](#_Toc489437295)

[**3.1. Efficacy criteria** 14](#_Toc489437296)

[3.1.1. Cytogenetic response criteria 14](#_Toc489437297)

[3.1.2. Molecular responses judgment 14](#_Toc489437298)

[**3.2. Trial termination criteria** 14](#_Toc489437299)

[**3.3. Special blood tests** 15](#_Toc489437300)

[3.3.1 Flow cytometry 15](#_Toc489437301)

[3.3.2 Bosutinib trough concentration 15](#_Toc489437302)

[4. Patient selection criteria 15](#_Toc489437303)

[**4.1. Eligibility criteria** 15](#_Toc489437304)

[4.1.1.Enrollment eligibility criteria 15](#_Toc489437305)

[**4.2. Exclusion criteria** 16](#_Toc489437306)

[5. Enrollment 16](#_Toc489437307)

[**5.1. Enrollment procedures** 16](#_Toc489437308)

[**5.2. Precautions in enrollment** 17](#_Toc489437309)

[6. Enrollment procedure and Trial plan 17](#_Toc489437310)

[**6.1. Enrollment procedure** 17](#_Toc489437311)

[**6.2. Administration dose and method** 17](#_Toc489437312)

[**6.3. Protocol termination or completion criteria** 18](#_Toc489437313)

[6.3.1. Definition of the protocol completion 18](#_Toc489437314)

[6.3.2. Protocol termination criteria 18](#_Toc489437315)

[**6.4. Concomitant and supportive therapies** 18](#_Toc489437316)

[6.4.1. Acceptable concomitant and supportive therapies 18](#_Toc489437317)

[6.4.2. Unacceptable concomitant and supportive therapies 19](#_Toc489437318)

[**6.5. Post-treatment** 19](#_Toc489437319)

[7. Expected adverse reactions 19](#_Toc489437320)

[**7.1. Assessment of adverse events and reactions** 19](#_Toc489437321)

[**7.2. Expected AEs** 19](#_Toc489437322)

[8. Endpoint, clinical assay, assessment schedule 20](#_Toc489437323)

[**8.1. Enrollment endpoint** 20](#_Toc489437324)

[**8.2. Testing and assessment during the treatment period** 20](#_Toc489437325)

[**8.3. Laboratory test schedule** 21](#_Toc489437326)

[**8.4. Safety endpoint conducted as needed** 21](#_Toc489437327)

[**8.5. Testing and assessment during the follow-up survey period** 21](#_Toc489437328)

[9. Data collection 21](#_Toc489437329)

[**9.1. Record form types and submission deadlines** 21](#_Toc489437330)

[**9.2. Method of sending Record forms** 22](#_Toc489437331)

[10. Reports of Aes 22](#_Toc489437332)

[10.1. AEs obligating a report 22](#_Toc489437333)

[10.1.1**. AEs obligating an immediate report** 22](#_Toc489437334)

[10.1.2. AEs that obligate standard report 23](#_Toc489437335)

[10.2. **Facility Chief Investigator's reporting obligation and reporting procedure** 23](#_Toc489437336)

[10.2.1. Secondary cancer 23](#_Toc489437337)

[10.2.2. Primary report 23](#_Toc489437338)

[10.2.3. Second report 23](#_Toc489437339)

[10.2.4. Tertiary report 23](#_Toc489437340)

[10.2.5. Standard report 23](#_Toc489437341)

[10.3. **Responsibilities of the Principal Investigator/Research Administrative Bureau** 24](#_Toc489437342)

[10.3.1. Judgement of the requirement of ceased enrollment and urgent report to the facility 24](#_Toc489437343)

[10.3.2. Reporting to the Efficacy and Safety Assessment Committee 24](#_Toc489437344)

[10.3.3. Examination of AEs in regular monitoring 24](#_Toc489437345)

[10.4. **Assessment by the Efficacy and Safety Assessment Committee** 24](#_Toc489437346)

[11. Efficacy Evaluation and Endpoint definition 25](#_Toc489437347)

[**11.1. Definition of the Full Analysis Set** 25](#_Toc489437348)

[11.1.1. All enrolled cases: Full analysis set (FAS) 25](#_Toc489437349)

[11.1.2. Per-Protocol Set (PPS) 25](#_Toc489437350)

[12. Statistical Analysis 25](#_Toc489437351)

[**12.1. Principal analysis and Criteria** 25](#_Toc489437352)

[**12.2. Scheduled Enrollment size・Enrollment period・Follow-up period** 25](#_Toc489437353)

[**12.3. Interim analysis and early termination of the trial** 26](#_Toc489437354)

[**12.4. Final analysis** 26](#_Toc489437355)

[13. Ethical considerations 26](#_Toc489437356)

[**13.1. Protection of patients** 26](#_Toc489437357)

[**13.2. Informed consent** 26](#_Toc489437358)

[13.2.1. Explanation to patients 26](#_Toc489437359)

[13.2.2. Explanation to patients 27](#_Toc489437360)

[**13.3. Protection of privacy and patient identification** 27](#_Toc489437361)

[**13.4. Protocol Compliance** 28](#_Toc489437362)

[**13.5. Facility Ethics committee (Approval by the Institutional Review Board (IRB)** 28](#_Toc489437363)

[**13.6. Annual update of the IRB Approval** 28](#_Toc489437364)

[**13.7. Modifications to protocol details** 28](#_Toc489437365)

[13.7.1. Amendments 28](#_Toc489437366)

[13.7.2. Revisions 28](#_Toc489437367)

[13.7.3. Memorandum 29](#_Toc489437368)

[13.7.4. Protocol amendments /Facility IRB approval at the time of amendment 29](#_Toc489437369)

[14. Research funding and conflicts of interest 29](#_Toc489437370)

[15. Monitoring and Auditing 29](#_Toc489437371)

[**15.1. Routine monitoring** 29](#_Toc489437372)

[**15.2. Items for monitoring** 30](#_Toc489437373)

[**15.3. Protocol deviation / violation** 30](#_Toc489437374)

[15.3.1. Violation 30](#_Toc489437375)

[15.3.2. Deviation 30](#_Toc489437376)

[15.3.3. Acceptable deviation 31](#_Toc489437377)

[**15.4. Facility on-site audits** 31](#_Toc489437378)

[16. Research organization 31](#_Toc489437379)

[**16.1. Research organization name** 31](#_Toc489437380)

[**16.2. Data and Safety Monitoring Board** 31](#_Toc489437381)

[**16.3. Chief Statistical analyst** 32](#_Toc489437382)

[**16.4. Data center** 32](#_Toc489437383)

[16.5**. Protocol Development Committee (In Japanese alphabetical order)** 32](#_Toc489437384)

[16.6. **Participating facilities and Facility chief investigator** 32](#_Toc489437385)

[17. Publication of research results 33](#_Toc489437386)

[18. References 33](#_Toc489437387)

APPENDIX1 Trial Participation Notice

APPENDIX2 Facility IRB Notice of Approval

APPENDIX3 Description・Consent form

APPENDIX4-1 Pre-enrollment form

APPENDIX4-2 Official Enrollment form

APPENDIX5-1 Pre-enrollment confirmation form

APPENDIX5-2 Official Enrollment confirmation form

APPENDIX6-1 Treatment period Case Record forms

APPENDIX6-2 Post-Termination Start Case Record forms

APPENDIX7-1 Terminational/Completion during Trial Period Report form

APPENDIX7-2 Trial Completion Report Form

APPENDIX8-1 Molecular Genetic Recurrence Follow-up Survey Form

APPENDIX8-2 Molecular Genetic Progress-free Follow-up Survey Form

APPENDIX9 AE/AR/ADR Report

APPENDIX10 Declaration of Helsinki (translated by the Japan Medical Association)

APPENDIX11 Drug information (drug package insert)

# 1. Purpose and Endpoint

## **1.1. Purpose**

When bosutinib is started at the standard dose of 500 mg, QD as the second or third line treatment or later to chronic myeloid leukemia in chronic phase (CML-CP) that have been resistant or intolerance to ABL tyrosine kinase inhibitors (TKIs) in the first line treatment, severe adverse effects (AEs) such as diarrhea or liver failure occur highly frequently, requiring suspension or termination of treatment. We will investigate whether the dose interruption or discontinuation of treatment can be lowered by starting bosutinib at the lower doses (200mg QD) and gradually titrating.

## **1.2. Endpoint**

### 1.2.1. Primary Endpoint:

Treatment drop-out rate due to AEs by 12 months after initiation of bosutinib

### 1.2.2. Secondary endpoints:

1. Rate of treatment interruptions
2. Mean bosutinib doses at 12 months after initiation of bosutinib
3. Administration period of bosutinib and its median dose intensity/relative dose intensity up to 12 months after initiation of bosutinib
4. Cumulative complete cytogenetic response (CCyR) maintenance rate at 6 and 12 months after initiation of bosutinib
5. Cumulative major molecular response (MMR) rate and cumulative deep molecular response (DMR rate) at 3, 6, 9 and 12 months after initiation of bosutinib
6. Incidence of all grades or grade 3 or 4 AEs

### 1.2.3. Exploratory endpoints:

1. Peripheral blood lymphocyte profiling
2. Bosutinib trough concentration, AEs and molecular responses

# 2. Background

## **2.1. Subjects**

### 2.1.1. Target disease

CML is a disease characterized by irreversible and unlimited increase of chiefly granulocytes due to cancerization of multipotent hematopoietic stem cells and is associated with the anomalous chromosomal abnormality of the Philadelphia (Ph) chromosome. *bcr-abl* fusion gene resulting from this chromosomal abnormality results in the transcription of the *bcr-abl* mRNA, and the translated BCR-ABL fused protein is understood to be the main cause of the disease. The annual incidence of CML in Japan is 1.5 per 100 thousand people, which is equal to those of Western countries, and the total number of patients is estimated to be approximately 12,000.

CML is a hematopoietic malignancy that progresses from the chronic phase (CP) to the accelerated phase (AP), and finally to the blast phase (BP). Although symptoms remain mild in the CP, lack of adequate treatment will lead the patient to the accelerated phase normally within several years, and eventually progress to BP characterized by increased blasts, or immature cells in the blood and bone marrow. BP is a lethal pathological condition that manifests severe symptoms such as bleeding, infections and anemia. Before effective treatments were available, the median survival period was 3-5 years.

### 2.1.2. Basis for setting the population

Bosutinib, which is one of the second generation TKIs, has demonstrated clinical efficacy in clinical trials (Phase I and II) when administered orally at a dose of 500mg QD to Ph^+^ CML patients with imatinib resistance/intolerance. Clinical trials demonstrated that its toxicity was mostly manageable (Cortes J et al. *JCO* 2012). Diarrhea (95%), rash (57%) and ALT elevation (38%) were reported as general AEs in P I/II clinical trials in Japanese. The trial protocols instructed suspension or dose reduction at 100mg increments based on the duration or severity of maintained toxicity; however, in a sub-analysis on cases of second line treatment, 27 cases (96%) were noted to require suspension or dose reduction due to adverse events. The dose intensity of bosutinib is 339.3 mg/day (228.4-500.0) and the relative dose-intensity is 0.68 (0.46-1.00), and there were 9 cases (32%) who ended in the termination of bosutinib after 3 or more steps of dose reduction during the clinical trial (Nakaseko, *et al. Int J Hematol* 2015).

The BELA trial compared the efficacy and safety of bosutinib and imatinib used for first line treatment for CML-CP. At 12 months, there was no significant difference between the two groups in the complete cytogenetic response (CCyR) rate (bosutinib group 70%，95%CI 64-76% vs. imatinib group 68%, 95% CI 62-74%, two-sided tests of statistical significance *P* = 0.601), thus the trial did not demonstrate the superiority of bosutinib as first line treatment (Gambacorti-Passerini C Et al. *Am J Hematol* 2014). Because of AEs, the drop-out rate of the bosutinib treatment group was unexpectedly high (48 cases, 19%), of which 15 cases (31%) dropped out after registered baseline data. That is, the early-stage drop-out cases in the bosutinib group were included as the low-response cases in the ITT analysis, which lowered the complete response (CR) rate of the bosutinib group, which may have thus eliminated the difference between the two groups.

The AEs of bosutinib include diarrhea, rash and ALT elevation, and the drug exhibits a different toxicity profile from imatinib. Diarrhea occurs most frequently 1-2 days after initiation of bosutinib, and its severity depended on the dose; thus, managing diarrhea in the early stages of introducing the drug is essential to prevent termination of bosutinib treatment. There are an increasing number of cases in which diarrhea can be managed through appropriate use of anti-diarrhetics; however, suspension or dose reduction remains the only countermeasure for liver failure, which frequently occurs as a clinical problem. As such, we decided to conduct a trial to gradually titrate the dose while controlling AEs in cases of CML-CP that manifested resistance or intolerance in prior TKI therapy.

## **2.2. Standard treatment**

The standard dosage of bosutinib is 500mg dose of QD (per os). The dose is gradually titrated according to the patient’s conditions, and can be increased up to 600mg QD.

## **2.3. Drugs used**

Bosutinib (Bosulif^®^ tablets)

Bosutinib is a new BCR-ABL TKI for CML and Ph^+^ acute lymphoblastic leukemia (ALL). Phase I trials have been started since 2004, which validated its safety and tolerance. Phase II trials were started in January 2006 on CML patients who exhibited imatinib resistance or intolerance, and international Phase III non-randomized open-label parallel group trials with imatinib as a control drug were started in February 2008 on untreated CML-CP patients. Results showed clinical utility as an effective method for second and subsequent line treatment. In Japan, Phase I trials started in 2006. Phase II trials started in December 2007 on CML patients who exhibited imatinib resistance or tolerance (second line treatment) and in January 2012, clinical trials on CML patients after imatinib treatment who exhibited resistance or intolerance to dasatinib or nilotinib as third line treatment. Japanese patients were also included in the international Phase II trial on untreated CML-CP patients. The results validated its efficacy and safety for second or subsequent treatment in Japanese CML patients, and manufacture and sales approval was granted in September 2014 for the indication of “CML with resistance or intolerance to the prior TKI treatment”.

## **2.4. Treatment regimen of the trial**

Oral bosutinib is introduced at 200mg QD for the initial dose. If all AEs are ≤ grade 2, the dose is gradually titrated by 100mg/day every two weeks. If any AEs are ≥ grade 3, the administration is suspended until the relevant AE returns to ≤ grade 1 and once it does, the administration is restarted from the decreased dose (100mg less than the last dose) (Ex: If the subject had a grade 3 adverse event at 300mg QD, the administration is suspended until the AE reduces to grade 1, and restarted from a 200mg QD. If any of the AEs are ≥ grade 3 at the initial dose of 200mg QD, the administration will restart at the same dose (i.e., 200mg QD) when the AE lowers to ≤ grade 1. When all AEs are maintained at ≤ grade 2, it is titrated again by 100mg/day every two weeks. This method of titration is continued until the daily dose reaches 500mg. When the same ≥ grade 3 AE is observed for two consecutive administrations at the same dose, a decreased dose (100mg less than that dose) is given for treatment as the maintenance dose. If the patient cannot take more than 300mg QD 3 months after initiation of bosutinib, this protocol is terminated. Furthermore, the protocol is terminated if a ≥ grade 3 AE cannot be improved to ≤ grade 1 for more than four weeks despite withdrawal, and is switched to method of treatment other than bosutinib. If it is judged as fail based on European LeukemiaNet (ELN) diagnosis criteria at 3, 6 and 12 months after starting treatment (efficacy criteria of TKI treatment for the secondary treatment on patients for whom imatinib treatment for **CML (ELN 2013 version**; Appendix Table 1), this protocol is terminated and the treatment is switched to something other than bosutinib. Subjects do not take bosutinib on the day of the visit, and after checking for any AEs by blood tests and clinical observations, the subject restarts treatment based on principal physician’s instructions at the dose prescribed by the protocol.

## **2.5. Trial design**

### 2.5.1. Basis for setting endpoint

Bosutinib, which is one of the second generation TKIs, has demonstrated enough efficacy and safety in the clinical trials (Phase I and II) when administered orally at a dose of 500mg QD to Ph^+^ CML patients with imatinib resistance/intolerance. The clinical trials also demonstrated that its toxicity was manageable (Cortes J et al. *JCO* 2013). In Japanese PI/II trials, diarrhea (95%), rash (57%) and ALT elevation (38%) were reported as general AEs. This trial protocol instructed interruption or dose reduction at 100mg increments based on the duration or severity of maintained toxicity; however, in a sub-analysis on cases of second line treatment, 27 cases (96%) were noted to require interruption or dose reduction due to AEs. The dose intensity of bosutinib is 339.3 mg/day (228.4-500.0) and the relative dose-intensity is 0.68 (0.46-1.00), and there were 9 cases (32%) who ended in the termination of bosutinib after 3 or more steps of dose reduction during the trial (Nakaseko, *et al. Int J Hematol* 2015).

### 2.5.2. Trial design


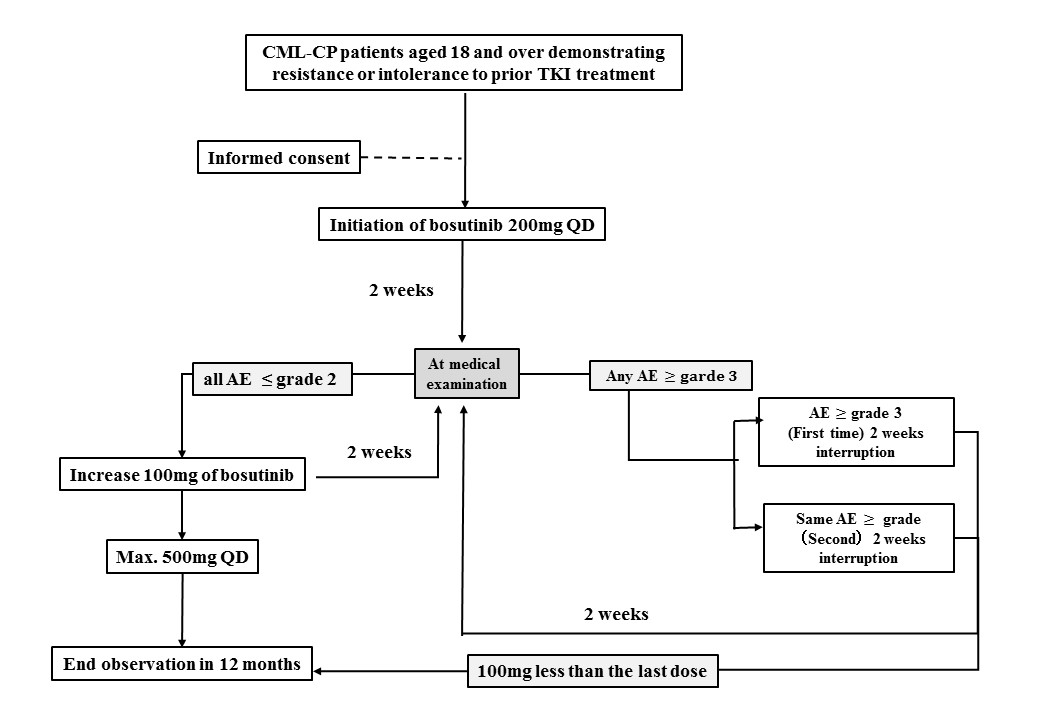


### 2.5.3. Clinical hypothesis and basis for setting enrolled sample

In the bosutinib Phase II Trial conducted in Japan, the drop-out rate of bosutinib treatment 12 months after switching to bosutinib from the previous treatment was 32% (Nakaseko C, *et al. Int J Hematol*. 2015). In the current trial, we set a drop-out rate equivalent to this as the null hypothesis，drop-out rate 14% as the alternative hypothesis and used SWOG statistical tools (<https://stattools.crab.org/>). The number of cases required at power 0.8 and one-sided significance level 5% was 33 cases. Considering protocol deviation, etc., we determined the official target enrolment sample size for this one-year trial as 35 people.

## **2.6. Summary of expected benefits and risks (disadvantages) from participating in the trial**

### 2.6.1. Expected benefits

Because bosutinib is started from a low dose, the possibility of onset of severe AEs may be lowered compared to the standard method of starting at 500mg QD. Reduced severity and decreased incidence of AEs may lower the rates of discontinuation during treatment or periods of discontinuation and increase the total dose during a given period. Considering the high rate of discontinuation that occur from initiation of bosutinib at the standard dose, we can expect higher efficacy by selecting the titration method. Furthermore, patients’ administration adherence is likely to be lower if they experience severe AEs in the early stages of initiation of bosutinib. Since this method starts with a low dose and gradually lets the body get used to it, the severity of AEs will be lowered, which reduces patients’ fear of the drug, and thus improving their adherence to the treatment and the treatment efficacy can be expected to improve as well.

All costs of the treatment during the trial will be paid by insurance or out of pocket by the participating patients; however, the testing costs of flow cytometry and bosutinib blood concentration levels will be covered by the researchers.

### 2.6.2. Expected risks and disadvantages

Since the treatment is started at a low dose, the amount of time it takes to reach effective dose is longer, which may delay the onset of treatment effects. This may induce leukemia cells that exhibit resistance to bosutinib.

## **2.7. Significance of this trial**

Starting the bosutinib dose at 500mg QD powerfully inhibits CML cells and can be expected to achieve deeper remission. However, gastrointestinal symptoms such as diarrhea or AEs such as liver function failures occur at high frequencies under 500mg QD dosage, thus requiring many cases to discontinue treatment. We believe it is important to investigate whether it is possible to achieve adequate treatment efficacy while preventing AEs by the titration protocol by starting treatment at low doses (200mg QD).

# 3. Criteria and definitions used in the present trial

## **3.1. Efficacy criteria**

**Judge the efficacy of second line TKI inhibitor on CML patients who failed prior TKI treatment at 3, 6, and 12 months after starting treatment on CML according to the ELN 2013.**

### 3.1.1. Cytogenetic response criteria

Measure Ph^+^ cell ratio by the FISH method.

### 3.1.2. Molecular responses judgment

The real-time quantitative polymerase chain reaction (RQ-PCR) conducted by a central laboratory (SRL or BML) will quantify *BCR-ABL* mRNA quantity according to the International Scale (IS).

## **3.2.** **Trial termination criteria**

If the first 10 enrolled patients have terminated at 3 months after starting the trial and if 4 subjects have terminated the protocol, this BOGI trial itself will be terminated in due consideration of ethics.

## **3.3. Special blood tests**

### 3.3.1 Flow cytometry

For flow cytometry, the measurements will be taken 3 total times, once at the time of pre-enrollment screening, and at 6 and 12 months after starting bosutinib treatment by central testing based on surface markers to calculate the real values. In order to standardize the time of blood sampling at 2 hours after administration, the subject will take bosutinib after visiting the hospital and have blood sampled 2 hours +/- 30 minutes (+/-30 minutes is considered permissible range) (The time between administration of bosutinib and blood sampling will be recorded.)

2 Color

・ CD3 × CD56 ・ CD8 × CD4 ・ CD57 × CD14

・ CD3 × CD8 ・ CD16 × CD56 ・ CD3 × CD57

・ CD56 × CD57

3 Color

・ CD4 × CD25 × CD127

### 3.3.2 Bosutinib trough concentration

At every visit, EDTA blood sampling tubes will be used to sample 2ml of blood before bosutinib administration (trough), and will be frozen for preservation after plasma separation. The 2 ml for EDTA blood sampling can be the blood remaining from the blood count. The time and dose of bosutinib taken the previous day and date and time of blood sampling will be recorded. If the trough cannot be confirmed, preservation will be skipped. Blood concentration measurement will be conducted by the US Inventive Health Clinic.

# 4. Patient selection criteria

Patients who satisfy all the following eligibility criteria and for whom none of the exclusion criteria applies will be considered patients eligible for enrollment.

## **4.1. Eligibility criteria**

### 4.1.1.Enrollment eligibility criteria

1. Patients of major BCR-ABL-positive CML-CP
2. Patients aged 18 or above at the time of pre-enrollment
3. Patients who exhibited resistance or intolerance to 1 or 2 other TKI
4. Patients with ECOG performance status 0-2
5. Patients whose function of the principal organs (liver, kidneys lungs) are maintained according to criteria by each institution
6. Patients whose written consent was obtained (the consent of parents or guardians required in the case of minors)

## **4.2. Exclusion criteria**

Patients cannot be considered subjects of this trial if any of the following criteria apply:

1. Patients who have history of taking anti-cancer drugs other than hydroxyurea for CML
2. Patients who are newly diagnosed CML
3. Patients who have progress to AP or BP
4. Patients with severe or uncontrollable complications
5. Patients with complications of inflammatory bowel disease
6. Pregnant and breastfeeding women, patients who wish to get pregnant within 12 months
7. Patients who are participating in another clinical trial
8. Patients with known T315I or V299L mutation
9. Concomitant medications known to be strong inducers or inhibitors of P450 isoenzyme CYP3A4
10. Known HIV and/or active viral hepatitis (hepatitis B or C)
11. Impaired cardiac function, including any of the following:

a. History of or presence of complete left bundle branch block, right bundle branch block plus left anterior hemiblock, bifascicular block in screening ECG

b. ST depression of >1mm in 2 or more leads and/or T wave inversions in 2 or more contiguous leads in screening ECG

c. Congenital long QT syndrome

d. QTc > 450 msec in the screening ECG

e. QT-prolonging concomitant medication

f. History of or presence of significant ventricular or atrial tachyarrhythmias in screening ECG

g. Myocardial infarction within 6 months prior to inclusion

h. Unstable angina diagnosed or treated during the past 12 months

i. uncontrolled hypertension, history of labile hypertension

# 5. Enrollment

## **5.1. Enrollment procedures**

Confirm that the patient meets all eligibility criteria and that none of the exclusion criteria apply and, fill all the required items on the Case Enrollment Form (APPENDIX4) and fax the case enrollment form to the data center.

For inquiries on patient enrollment and reception hours

Data center: Noriko Yoshida

Clinical Research Center, Saga University Hospital

5-1-1, Nabeshima, 849-8501 Saga, Japan

TEL: 0952-34-3400, FAX: 0952-34-2085

E-mail: nyoshida@cc.saga-u.ac.jp

For inquiries on the test such as patient selection criteria:

Research Administration Bureau: Shinya Kimura

Division of Hematology, Respiratory diseases and Oncology,

Department of Internal Medicine, Faculty of Medicine, Saga University

5-1-1, Nabeshima, 849-8501Saga, Japan

TEL: 0952-34-2366, FAX: 0952-34-2017

E-mail: shkimu@cc.saga-u.ac.jp

## **5.2. Precautions in enrollment**

- 1. All enrollments after starting protocol treatment cannot be accepted, without exceptions.
  2. If there are missing items from the case enrollment form, the enrollment cannot be received until all items are met.
  3. Once enrolled, the “Case Enrollment Confirmation Form” (APPENDIX5) will be faxed back to the physician in charge or the facility coordinator from the data center. Please conserve this form.
  4. Once enrolled, a patient’s enrollment cannot be cancelled (their file will not be deleted from the database. In cases of duplicate enrollment, the enrollment data from the first enrollment (the enrollment number) will be used.
  5. If erroneous or duplicate enrollment is discovered, please contact the data center promptly.

# 6. Enrollment procedure and Trial plan

## **6.1. Enrollment procedure**

If the attending physician judges that the patient meets entry criteria, the physician will send an enrollment request form to the administrative bureau.

## **6.2. Administration dose and method**

Oral bosutinib is introduced at 200mg QD for the initial dose. If all AEs are ≤ grade 2, the dose is gradually titrated by 100mg/day every two weeks. If any AEs are ≥ grade 3, the administration is suspended until the relevant AE returns to ≤ grade 1 and once it does, the administration is restarted from the decreased dose (100mg less than the last dose) (Ex: If the subject had a grade 3 adverse event at 300mg QD, the administration is suspended until the AE reduces to grade 1, and restarted from a 200mg QD. If any of the AEs are ≥ grade 3 at the initial dose of 200mg QD, the administration will restart at the same dose (i.e., 200mg QD) when the AE lowers to ≤ grade 1. When all AEs are maintained at ≤ grade 2, it is titrated again by 100mg/day every two weeks. This method of titration is continued until the daily dose reaches 500mg. When the same ≥ grade 3 AE is observed for two consecutive administrations at the same dose, a decreased dose (100mg less than that dose) is given for treatment as the maintenance dose. If the patient cannot take more than 300mg QD 3 months after initiation of bosutinib, this protocol is terminated. Furthermore, the protocol is terminated if a ≥ grade 3 AE cannot be improved to ≤ grade 1 for more than four weeks despite withdrawal, and is switched to method of treatment other than bosutinib. If it is judged as fail based on European LeukemiaNet (ELN) diagnosis criteria at 3, 6 and 12 months after starting treatment (efficacy criteria of TKI treatment for the secondary treatment on patients for whom imatinib treatment for **CML (ELN 2013 version**; Appendix Table 1), this protocol is terminated and the treatment is switched to something other than bosutinib. Subjects do not take bosutinib on the day of the visit, and after checking for any AEs by blood tests and clinical observations, the subject restarts treatment based on principal physician’s instructions at the dose prescribed by the protocol.

## **6.3. Protocol termination or completion criteria**

### 6.3.1. Definition of the protocol completion

The date of protocol completion is 12 months after initiation of bosutinib.

### 6.3.2. Protocol termination criteria

The protocol will be terminated in the case of any of the following:

1. Loss of hematological remission, loss of cytogenetic remission, loss of molecular responses, death
2. Withdrawal of consent
3. Protocol violation or if ineligibility is discovered
4. If hematopoietic cell transplant is possible, and this selection is made
5. If the subject cannot tolerate ≥ 300mg QD 3 months after starting treatment, then this protocol will be interrupted. If an adverse event ≥ grade 3 cannot be improved after four or more weeks to ≤ grade 1 despite taking a suspension, this protocol will similarly be interrupted and the treatment would be switched to something other than bosutinib.
6. If ELN (2013 version) diagnosis criteria at 3, 6 and 12 months after initiation of bosutinib judged that (efficacy criteria of TKI treatment for the second line treatment and after on patients for whom imatinib treatment for **CML failed (ELN 2013 version;** Failure according to Appendix Table 1)
7. If the subject becomes pregnant
8. If the physician in charge judges that termination is required

## **6.4. Concomitant and supportive therapies**

### 6.4.1. Acceptable concomitant and supportive therapies

Drugs for the purpose of treating complications and AEs such as antibiotics, analgesics, transfusions and G-CSF formulations may be used as appropriate if the concomitant use is considered valid. Particularly, we recommend the prophylactic administration of antiflatulents before starting the protocol treatment, and oral antidiarrhetics such as loperamide can be prescribed as needed. However, the reason for use, treatment method, drug name, dose, administration method and period must be written into the case report for concomitant use.

### 6.4.2. Unacceptable concomitant and supportive therapies

During the protocol treatment, all treatment for CML other than the drug used in this study are prohibited. All chemical therapies other than bosutinib, immunotherapy, radiotherapy and other treatments that may affect the evaluation of this trial will be avoided. All concomitant treatment with trial drugs (including drugs that are not anti-cancer drugs) will also be avoided.

Health supplement foods and vitamin and mineral supplements will not be regulated. However, concomitant use of herbs, etc., which increases physiological activity or affect drug metabolism, or mushrooms that are given as alternative medicine are also avoided.

## **6.5. Post-treatment**

There are no particular regulations on “post-treatment” given after termination of bosutinib according to the protocol termination criteria.

# 7. Expected adverse reactions

## **7.1. Assessment of adverse events and reactions**

CTCAE ver4.0* will be used for assessment of adverse events and reactions. In grading AEs, the grade that fits most closely to the respective definitions of grades 1-5 will be applied. For considering the causal relationships between death and the adverse events observed in the treatment-related death will be written in the Treatment Completion Report Form, etc., and the report will be sent immediately. The grade that is written in the records form will always be noted in the patient charts as well.

*Japanese version of the CTCAE *ver.* 4.0 (<http://www.jcog.jp/doctor/tool/CTCAEv4J_20100911.pdf>) Grade 1 Mild; have no or mild symptoms; based on clinical or test findings only; requires no treatment

Grade 2 Moderate; requires minimal/local/non-invasive treatment; limits to personal activities of daily living appropriate for the patient’s age

Grade 3 Severe or is medically serious, but poses no immediate threat to survival; requires hospitalization or extension of the period of hospitalization; incapable of activity/movement; limits to movements for personal activities of daily living

Grade 4 Threatens survival; requires urgent treatment

Grade 5 Death due to AE

## **7.2. Expected AEs**

See “APPENDIX11 Drug Information (drug package insert)” for expected drug AEs in this trial.

# 8. Endpoint, clinical assay, assessment schedule

## **8.1. Enrollment endpoint**

- 1. Physiological findings, general condition: PS, subjective and objective symptoms
  2. Peripheral blood count: leukocyte count, differential count of leukocytes, hemoglobin, erythrocyte count, thrombocyte count
  3. At screening: AMP-CML, *BCR-ABL* mRNA RQ-PCR, Nested PCR (at each institution)
  4. At time of official enrollment: *BCR-ABL* mRNA RQ-PCR (at the Central Laboratory)
  5. Lymphocyte analysis with flow cytometry (at time of screening)

## **8.2. Testing and assessment during the treatment period**

- 1. Physiological findings, general condition: PS, subjective and objective symptoms (once/month)
  2. Peripheral blood count: leukocyte count, differential count of leukocytes, hemoglobin, erythrocyte count, thrombocyte count. In general, the attached document will be followed and conducted every week to 2 months after starting administration, and once a month thereafter
  3. *BCR-ABL* mRNA RQ-PCR (once/month at the Central Laboratory)
  4. Lymphocyte analysis with flow cytometry (at 6 and 12 months)
  5. Bosutinib trough concentration (at every visit)

## **8.3. Laboratory test schedule**

|  | screening | Registration | Initiation of treatment | Every week until 2 months,  Thereafter once a month | 6 months | 7 months  ～11 months | 12 months | termination |
| --- | --- | --- | --- | --- | --- | --- | --- | --- |
| eligibility | ○ |  |  |  |  |  |  |  |
| Informed consent |  | ○ |  |  |  |  |  |  |
| Age/sex/diagnosis/day of first visit | ○ |  |  |  |  |  |  |  |
| Prior TKI treatment | ○ |  |  |  |  |  |  |  |
| Complication | ○ |  |  |  |  |  |  |  |
| Treatment other than TKI | ○ | ○ |  |  |  |  |  |  |
| Physical examination, PS | ○ | ○ | ○ | ○ | ○ | ○ | ○ | ○ |
| PB count |  | ○ | ○ | ○ | ○ | ○ | ○ | ○ |
| Trough concentration |  |  |  | ○ |  |  | ○ |  |
| BCR-ABL mRNA (IS) |  | ○ | ○ | ○ | ○ | ○ | ○ | ○ |
| FCM |  | ○ |  |  | ○ |  | ○ |  |

## **8.4. Safety endpoint conducted as needed**

- 1. In case of dyspnea: BNP, arterial blood gas analysis, chest radiography, ECG
  2. In case of arrhythmia: twelve-lead resting ECG, ECG
  3. In case of suspected abnormal renal function: urine test (spot urine: urine glucose, urine protein)
  4. Blood biochemistry

## **8.5. Testing and assessment during the follow-up survey period**

Survival, presence of adverse events, confirmation of recurrence

# 9. Data collection

## **9.1. Record form types and submission deadlines**

Record forms (Case Report Form: CRF) used in this trial and their submission deadlines are as follows.

Case Enrollment Form (APPENDIX4: at time of enrollment (See 5. Enrollment)

Case Data Record forms (APPENDIX5: within 4 weeks of starting bosutinib treatment)

Treatment Completion Report Form (APPENDIX6: within 2 weeks of terminating/completion of treatment)

The Case Enrollment Form is distributed before starting the trial, in advance to each facility from the data center together with the enrollment set, separately from the protocol. The Treatment Completion Report Form is mailed from the data center after enrollment.

## **9.2. Method of sending Record forms**

Pre-trial record forms are sent by fax. All CRF or Record forms during the trial are in general sent to the data center by mail, with the exception of the Case Enrollment Form. Enrollment Eligibility Confirmation and Enrollment Confirmation Notice at the time of enrollment will be sent exceptionally by fax because it is time-sensitive.

# 10. Reports of Aes

## 10.1. AEs obligating a report

### 10.1.1**. AEs obligating an immediate report**

Any of the following adverse events obligate an immediate report:

- 1. Threatens survival
  2. Ends in death
  3. Requires hospitalization or extension of a patient’s hospitalization duration
  4. Leaves permanent or marked damage or functional failure
  5. Causes congenital abnormalities or defects
  6. Judged to expose the subject to risk by the facility chief investigator
  7. Requires internal or surgical intervention to prevent any of the above events
  8. Exposure during pregnancy
  9. Exposure while breastfeeding
  10. Occupational exposure
  11. Lack of drug effect (only in cases of death due to disease progress during the period subject to reporting of this research)
  12. Hy's Law Cases
- Patients whose AST or ALT baseline values were within standard range, but AST elevated to more than 3 times the upper limit of standard values, or more than 2 times the upper limit of standard values of total bilirubin were found for both AST and ALT, without hemolysis, and alkaline phosphatase levels were less than two times the upper limit of standard values or unknown
- Patients whose AST or ALT baseline values already exceeded standard range, then increased to two times the baseline or three times or more than the upper limit of standard values, or increased to 8 times or more of the upper limit of standard values (determine with the lower of the two). At the same time, total bilirubin was more than two times the upper limit of standard values and increased from baseline value to amount equal to the upper limit of standard value, or more than 3 times the upper limit of standard value (determine with the lower of the two), without hemolysis, and alkaline phosphatase is less than two times the upper limit of standard value or unknown

### 10.1.2. AEs that obligate standard report

Any of the AEs below are subject to standard reporting

1. Expected Grade 4 toxicity. Grade 4 toxicity written in "7. Drug information/ Severe drug adverse reactions." Note that expected but severe adverse events are subject to standard report.
2. Unexpected Grade 2 or Grade 3 adverse events. Adverse events equivalent to Grade 2-3 that are not written in "7. Drug information/Principal drug adverse reactions."
3. Permanent or marked damage. Aplastic anemia, myelodysplastic syndrome, second primary cancer, etc.
4. Other serious medical events. Adverse events that do not fit under 10.1.1 2, 10.1.2 1-4, but information judged to be important for sharing with the chief investigator and research group.

## 10.2. **Facility Chief Investigator's reporting obligation and reporting procedure**

### 10.2.1. Secondary cancer

If an AE that is subject to Expedited reporting occurs, the physician in charge will promptly notify the facility chief investigator. If the facility chief investigator cannot be contacted, the physician in charge must take over the responsibilities of the facility chief investigator.

### 10.2.2. Primary report

The facility chief investigator must write the prescribed items into the "AE/AR/ADR Expedited Primary Report"(APPENDIX9) within 24 hours of learning about the occurrence of the adverse event, contact the data center by fax and phone as well as fax to Pfizer Inc.

### 10.2.3. Second report

Furthermore, the facility investigator notes the prescribed items to the ○ in the Secondary Report to write in more detailed information "AE/AR/ADR Expedited Primary Report"(APPENDIX9) than the aforementioned Case Report (A4: Create an attachment in free format, and send both to the data center by fax within 7 days of learning about the adverse event. In order to prioritize rapid expedition of the information, it is not serious if there are missing items or items that are unconfirmed in the report.

### 10.2.4. Tertiary report

Write the prescribes items that were left incomplete in the secondary report to the "AE/AR/ADR Expedited Primary Report"(APPENDIX9 circle "Tertiary Report") and send to the data center by fax within 7 days.

### 10.2.5. Standard report

The facility chief investigator writes the prescribed items in "AE/AR/ADR Expedited Primary Report"(APPENDIX9 circle "Standard Report") and fax to the data center by fax within 15 days of learning about the adverse event.

## 10.3. **Responsibilities of the Principal Investigator/Research Administrative Bureau**

### 10.3.1. Judgement of the requirement of ceased enrollment and urgent report to the facility

The principal researcher who gets the report from the facility chief investigator judges the urgency of the reported details, importance and degree of important, etc., and considers temporary suspension of enrollment as needed (contact the Enrollment center, data center and all participating facilities) or considers urgent communication that all participating facilities should know. It is possible to contact the enrollment center, data center or facilities by phone depending on the level of urgency; however, it should be followed as promptly as possible in writing (FAX, mail or email).

### 10.3.2. Reporting to the Efficacy and Safety Assessment Committee

The principal researcher reports to the Efficacy and Safety Assessment Committee within 15 days of learning about the AE if he or she judges that the AE reported by the facility via Expedited or standard reporting is a "10.1 AE obligating a report. At the same, he or she requests for an opinion of the principal researcher on the adverse event and a review of the validity of the response to the AE.

At this time, the results of investigation or measures (including judgment to continue or discontinue the trial) as the primary researcher should be included in the "AE/AR/ADR Expedited Primary Report" (APPENDIX9) and "AE/AR/ADR Report” (APPENDIX9) sent from the facility. Furthermore, for deaths that count for 10.1.1.1 or 10.1.2 1 that are judged to be treatment-related death, and expected severe AEs that count for 10.1.2.4, in addition to the individual patient's progress, if the incidence was judged to exceed the expected range, write this in the "AE/AR/ADR Report" (APPENDIX9) as well.

### 10.3.3. Examination of AEs in regular monitoring

In regular monitoring, the principal researcher/research administrative bureau considers the AEs report in the monitoring report created by the data center carefully, and checks that there is no missing information from the facility. They also check that the reported adverse events are all listed up in the regular monitoring report. Missing reports are to be clearly written in the group's assessment results report for the regular monitoring report.

## 10.4. **Assessment by the Efficacy and Safety Assessment Committee**

The Efficacy and Safety Assessment Committee follows the procedures related to clinical safety information to review and assess the reported details, and advises the principal researcher and facility investigator in writing on whether or not to continue the enrollment, and other future measures including whether or not revisions to the protocol are needed.

# 11. Efficacy Evaluation and Endpoint definition

## **11.1. Definition of the Full Analysis Set**

### 11.1.1. All enrolled cases: Full analysis set (FAS)

Of the patients enrolled according to "5.1 Enrollment Procedures," the set that excludes duplicate or erroneous enrollments is defined as "All enrolled cases."

Of All enrolled cases, all patients who meet the patient selection criteria defined in the protocol and for whom all or part of the protocol treatment was given after enrollment is the "Maximum FAS." This "Maximum FAS" is the principal FAS for efficacy and safety.

Whether they meet patient selection criteria is determined by the research administrative bureau. That is, cases that are only judged by the physician in charge and facility chief investigator will not be used. The final analysis requires approval of the chief investigator. For interim analysis, the data center may do the regular monitoring or reports at conferences before submitting the final analysis report on the maximum FAS with the approval of the research administrative bureau according to its judgment.

### 11.1.2. Per-Protocol Set (PPS)

For the purpose of considering the robustness of the analysis results of the FAS, we will define the "Per-Protocol Set." The PPS is the FAS minus patients who made serious protocol violations. Whether or not they made serious violations of the protocol will be determined by the Research Administrative Bureau andwill be approved by the chief investigator at the time of final analysis.

# 12. Statistical Analysis

## **12.1. Principal analysis and Criteria**

In the bosutinib Phase I/II Trial conducted in Japan, the drop-out rate due to AEs of bosutinib treatment by 6 months after switching to bosutinib from the previous treatment was 25% (Nakaseko C, *et al. Int J Hematol*. 2015). In the current trial, we set a drop-out rate due to AEs equivalent to this as the null hypothesis，drop-out rate due to AEs 9% as the alternative hypothesis and used SWOG statistical tools (<http://www.swogstat>. org/statoolsout.html). The number of cases required at power 0.8 and one-sided significance level 5% was 33 cases. Considering protocol deviation, etc., we determined the official target enrolment sample size for this one-year trial as 35 people.

## **12.2. Scheduled Enrollment size・Enrollment period・Follow-up period**

Scheduled enrolment sample size: 35 for formal enrollment

Enrollment period：One year from the date of approval by the ethics committee

Follow-up period: 12 months

Total length of study: 24 months

## **12.3. Interim analysis and early termination of the trial**

If the first 10 enrolled cases have terminated at 3 months after starting the trial and if 4 subjects have terminated the protocol, this BOGI trial itself will be terminated in due consideration of ethics.

## **12.4. Final analysis**

After completion of the Follow-up period, and after confirmation of data with the final survey, analysis will be conducted all endpoints. During all other periods, no analysis on the primary endpoint and secondary endpoints related to efficacy will not be conducted, with the exception of situations in which permission is given by the Efficacy and Safety Assessment Committee.The results of the final analysis will be put together by the data center as the “Final Analysis Report”, and will be submitted to the Research Administrative Bureau and the chief investigator.

# 13. Ethical considerations

## **13.1. Protection of patients**

All researchers involved in this trial will protect the rights and welfare of patients based on the newest version of the Declaration of Helsinki (revised October 2013), and adhere strictly to the Ministry of Education and Ministry of Health, Welfare and Labour’s “Ethical Guidelines for Medical and Health Research Involving Human Subjects” (effective April 1, 2015) and the protocol to conduct the trial.

## **13.2. Informed consent**

### 13.2.1. Explanation to patients

Before pre-enrollment, the physician in charge gives the Description of the Representative (APPENDIX3) or the descriptive document approved by the facility IRB to the patient, and explains the following orally in detail:

1. That this trial is a clinical trial

Difference between a Clinical trial and Clinical practice

1. Methods of the trial
2. Expected clinical risks and benefits
3. Item on the presence or absence of other treatments on said patient and expected important risks and benefits related to the treatment
4. In continuing to participate in the trial, if information that may impact the intentions of the subject, they will be promptly transmitted to the subject
5. Participation in the trial is by free will of the subject, that the subject can refuse or withdraw from participation in the trial at all times. Furthermore, they will not be subjected to disadvantageous treatment or lose benefits that should be given in the case that they do not participate in the trial will not be lost from refusal or withdrawal.
6. Protection of human rights

The maximum effort will be made to protect names or personal information.

1. Contacts for a medical facility or responsible physician in the case that health damage occurs
2. Incurred costs and compensation

Costs associated with the treatment will be paid by the insurance system, that compensatory treatment in the case of health damage will be made in general clinical practice, and will be same as general clinical practice.

1. Secondary use of data

Data may be used for secondary uses without linking to information that could identify individuals, with the exception of cases that are approved by the chief investigator’s facility IRB (possible use for meta-analysis, etc.)

1. Financial resources related to this clinical trial, possible conflicts of interest, relationships with researchers and health related organizations
2. Funding for this trial, conflicts of interest
3. Freedom of inquiry

Explain that they can freely inquire about the trial or details of the treatment.

### 13.2.2. Explanation to patients

Explain about the trial before pre-enrollment. Confirm that the patient has understood the details of the trial well, the request for participation in the trial. If the patient him or herself consents to participation in the trial, use the attached table Consent Form (APPENDIX3), write the name of the physician who explained and the name of the patient who received the explain and consented, the date that the consent was obtained, and have each sign by themselves.

Make two copies of the consent form. Hand one copy to the patient him or herself. The facility principal investigator keeps one copy. The original is conserved in the patient file.

## **13.3. Protection of privacy and patient identification**

The data center will not be informed of the names of the enrolled patient by the participating facility. Identification or referral of the enrolled patient will be done by the case enrolment number issued at enrolment, patient initials, birthdate, and patient file number. Data that allows a third party to find patient names, etc. or otherwise identify the patient directly without contacting a staff at the facility or illegally accessing the database will not be registered on the data center’s database. Correspondence of patient data between the facility, data center, and research administrative bureau will be done by mail or directly in person with the exception of case enrolment.

As a sample entry for Private Information Protection Law, the last four digits in the case of patient file number or an id number unique to the facility may be used. In the case of birth dates, write the year and month only. The patient initials are optional. (However, the case must be identifiable within the facility.)

## **13.4. Protocol Compliance**

Researchers participating in this trial will comply with this protocol as long as the patient’s safety and human rights are not violated.

## **13.5. Facility Ethics committee (Approval by the Institutional Review Board (IRB)**

In participating in this trial, the protocol and explanation form to the patient are approved by the ethics committee (Institutional Review Board: IRB) of the participating facility or by the Central Site Management Organization of the facility that functions as the main facility, and must be approved by the directors of the conducting facilities. If approval can be obtained, the chief investigator of each facility faxes a copy of the approval form and the Tracing Report (APPENDIX2) to the data center.

In the case of oral approval (if there is no IRB Approval Form) fill only the IRB Tracing Report and fax.

## **13.6. Annual update of the IRB Approval**

Each facility’s ethics committee or IRB’s annual update will be determined according to the regulations of each participating facility.

## **13.7. Modifications to protocol details**

Modifications to protocol details will be treated in two types of amendments and revisions. Additional descriptions that do not count as modifications to protocol details will be differentiated as a memorandum. They will be treated as specified below.

### 13.7.1. Amendments

Modifications related to changes that have the possibility of increasing risks for the patient participating in the trial, or partial modifications of the protocol related the primary endpoint of the trial.

Requires the approval of the group representative, and review and approval of the Efficacy and Safety Assessment Committee and each facility’s IRB. Write the approval date of the Efficacy and Safety Assessment Committee on the cover page.

### 13.7.2. Revisions

Modifications to the protocol that have no possibility of increasing risks for the patient participating in the trial, and are unrelated to the primary endpoint.

Review of the Efficacy and Safety Assessment Committee is not required. However, the approval of the group representative and report to the Efficacy and Safety Assessment Committee are required. Trials managed by the data center require approval by the data center director. Review and approval by the facility IRB are made according to the rules at each facility. It is also necessary to report to the chief investigator and administration bureau.

### 13.7.3. Memorandum

Supplementary descriptions of the protocol to be distributed by the research representative/research administrative bureau to parties concerned not for the purpose of modifying protocol details, but for the purpose of reducing discrepancies due to interpretation of writing, especially for alert precautions. Does not require review by the Efficacy and Safety Assessment Committee. Requires approval by the group representative and reporting to the Efficacy and Safety Assessment Committee. Requires approval of the data center director in the case of trials managed by the data center. Does not requiring writing in the cover page. Follow the rules of each facility for review and approval by the facility IRB.

### 13.7.4. Protocol amendments /Facility IRB approval at the time of amendment

In the case that amendments are made to the protocol or explanation document to the patient with the approval of the Data and Safety Monitoring Board during the trial, the amended protocol and explanation document must be approved by each facility’s ethics review committee (or IRB). If the modifications are not amendments but revisions, whether or not it requires review and approval by the ethics review committee (or IRB) will be determined according to the rules of each institution.

If IRB approval to the amendment is obtained, the facility chief investigator sends a copy of the IRB approval form to the data center. The original of the IRB approval form is conserved by the facility chief investigator and the copy is conserved by the data center.

# 14. Research funding and conflicts of interest

This research is to be conducted by the principal investigator with funding of Pfizer Inc. This trial uses a drug that is manufactured and sold by Pfizer Inc., but here we confirm that this does will not have any influence on the results of the research. Therefore, there are not “potential conflicts of interest” that may affect the results of the research and interpretation thereof in the planning, conduction and report of this research. We also confirm that conducting the research will not undermine the rights or benefits of tester.

# 15. Monitoring and Auditing

## **15.1. Routine monitoring**

Monitoring consists of the central monitoring based on the data written in the Case Record Forms (CRF) collected by the data center, and in-house monitoring, and will be assessed by the research administrative bureau, Drug and Safety Monitoring Board and principal investigator.

Monitoring Manager: Noriko Yoshida

Clinical Research Center, Saga University Hospital

5-1-1, Nabeshima, 849-8501 Saga, Japan

TEL: 0952-34-3400, FAX: 0952-34-2085

E-mail: nyoshida @cc.saga-u.ac.jp

## **15.2. Items for monitoring**

1. Patient enrolment status: Number of enrolled cases－Cumulative/by each period, all facilities /by each facility
2. Eligibility: Ineligibility /Cases that may have possibility of ineligibility: Facility
3. In protocol treatment /by treatment completion, reason for termination/completion: facility
4. Pre-treatment background factors: All enrolled cases
5. Adverse reactions / adverse events：All enrolled cases
6. Progression-free survival period：All enrolled cases
7. Other problems related to trial progress or safety

## **15.3. Protocol deviation / violation**

Protocol deviations are defined as cases in which drug administration, clinical assays or assessment of toxicity or efficacy, etc., are done without following the protocol. For monitoring, as a general rule, deviations that exceed a certain permissible range determined for each trial by the data center and principal investigator/ Chief Administration Bureau will be listed on the Monitoring Report as a “Case that may be deviations” and will be classified into one of the following after assessment by the research administrative bureau and the research group.

### 15.3.1. Violation

As a general rule, any of the deviations from the protocol rules below will be defined as a “violation.”

1. Influences the assessment of the trial endpoint.
2. The physician in charge or the facility is at cause.
3. Intentional or systematic.
4. Dangerous or the degree of deviation is remarkable.
5. Clinically inappropriate.

As a general rule, individual violations will be described for publication in papers for “Violations.”

### 15.3.2. Deviation

Deviations that neither 15.3.1 Violations nor 15.3.3 Acceptable deviation do not apply. To be mentioned for publishing the paper when a particular deviation is observed frequently.

### 15.3.3. Acceptable deviation

Deviation from the protocol within the permissible range established for each trial in advance or after the trial by the research group, or between the principal investigator/research administrative bureau and data center. Deviations within the pre-established permissible range will not be mentioned in the Monitoring Report.

## **15.4. Facility on-site audits**

As a general rule, no facility on-site audits will be conducted. Central monitoring will verify if the trial is conducted safely and according to the protocol.

# 16. Research organization

## **16.1. Research organization name**

BOGI Study Group

Co-Principal Investigator/owner：Shinya Kimura, MD, PhD.

Division of Hematology, Respiratory Medicine and Oncology,

Department of Internal Medicine, Faculty of Medicine, Saga University

5-1-1 Nabeshima, 849-8501 Saga, Japan

TEL: 0952-34-2366, FAX: 0952-34-2017

E-mail: [shkimu@cc.saga-u.ac.jp](mailto:shkimu@cc.saga-u.ac.jp)

Co-Principal Investigator：Naoto Takahashi, MD, PhD.

Department of Hematology, Nephrology, and Rheumatology,

Akita University Graduate School of Medicine

1-1-1 Hondo, 010-8543 Akita, Japan

TEL: 018-884-6111, FAX: 018-836-2613

E-mail: naotot@doc.med.akita-u.ac.jp

Research Administration Bureau：Shinya Kimura, MD, PhD.

Division of Hematology, Respiratory Medicine and Oncology,

Department of Internal Medicine, Faculty of Medicine, Saga University

5-1-1 Nabeshima, 849-8501 Saga, Japan

TEL: 0952-34-2366, FAX: 0952-34-2017

E-mail: shkimu@cc.saga-u.ac.jp

## **16.2. Data and Safety Monitoring Board**

Hisashi Gondo

Department of Hematology, Saga-ken Medical Centre Koseikan 1 Chome-12-9 Mizugae, 840-0054 Saga, Japan

TEL：0952-24-2171, FAX：0952-29-9390

Sano Masayuki

Department of Internal Medicine, Saga Prefectural Fujiyamato Onsen Hospital

1721-1 Fujichō Ōaza Umeno, 840-0516 Saga, Japan

TEL：0952-63-0111, FAX：0952-51-0138

## **16.3. Chief Statistical analyst**

Professor Atsushi Kawaguchi

Section of Clinical Cooperation System, Faculty of Medicine, Saga University,

5-1-1, Nabeshima, 849-8501 Saga, Japan

TEL: 0952-34-3400 FAX: 0952-34-2085

E-mail: akawa@cc.saga-u.ac.jp

## **16.4. Data center**

Noriko Yoshida

Clinical Research Center, Saga University Hospital

5-1-1, Nabeshima, Saga, Saga-ken, 〒849-8501

TEL: 0952-34-3400 FAX: 0952-34-2085

E-mail: nyoshida@cc.saga-u.ac.jp

## 16.5**. Protocol Development Committee (In Japanese alphabetical order)**

Professor Atsushi Kawaguchi Section of Clinical Cooperation System, Faculty of Medicine, Saga University

Professor Shinya Kimura Saga University Hospital Division of Hematology, Respiratory Medicine and Oncology

Professor Naoto Takahashi Akita University Graduate School of Medicine, Department of Hematology, Nephrology and Rheumatology

Associate Professor Hisako Yoshida Clinical Research Center, Saga University Hospital

## 16.6. **Participating facilities and Ffacility chief investigator**

Professor Shinya Kimura Saga University Hospital Division of Hematology, Respiratory Medicine and Oncology

Professor Naoto Takahashi Akita University Graduate School of Medicine, Department of Hematology, Nephrology and Rheumatology

Dr Takayuki Ikezoe Fukushima Medical University, Department of Hematology

Assistant Professor Jun Imagawa University Hospital, Department of Hematology and Oncology, Hiroshima, Radiation Biology Research Center

# 17. Publication of research results

Papers related to this research will be published by the participating facilities (facilities that had enrolled cases, and written in the order of larger enrollments or by joint names by small groups). We can predict that there will be several conference presentations, and will be determined upon discussion with each facility chief investigator. As a general rule, however, it will be in order of facilities with larger enrollment, or we will prioritize small groups in a round-robin.

# 18. References

1. Cortes JE, Kim DW, Kantarjian HM, Brümmendorf TH, Dyagil I, Griskevicius L, Malhotra H, Powell C, Gogat K, Countouriotis AM, Gambacorti-Passerini C. Bosutinib versus imatinib in newly diagnosed chronic-phase chronic myeloid leukemia: results from the BELA trial. J Clin Oncol 30: 3486-3492, 2012.

2. Gambacorti-Passerini C, Cortes JE, Lipton JH, Dmoszynska A, Wong RS, Rossiev V, Pavlov D, Gogat Marchant K, Duvillié L, Khattry N, Kantarjian HM, Brümmendorf TH. Safety of bosutinib versus imatinib in the phase 3 BELA trial in newly diagnosed chronic phase chronic myeloid leukemia. Am J Hematol 89: 947-953, 2014.

3. Brümmendorf TH, Cortes JE, de Souza CA, Guilhot F, Duvillié L, Pavlov D, Gogat K, Countouriotis AM, Gambacorti-Passerini C. Bosutinib versus imatinib in newly diagnosed chronic-phase chronic myeloid leukaemia: results from the 24-month follow-up of the BELA trial.　Br J Haematol 168: 69-81, 2015.

4. Nakaseko C, Takahashi N, Ishizawa K, Kobayashi Y, Ohashi K, Nakagawa Y, Yamamoto K, Miyamura K, Taniwaki M, Okada M, Kawaguchi T, Shibata A, Fujii Y, Ono C, Ohnishi K. A phase 1/2 study of bosutinib in Japanese adults with Philadelphia chromosome-positive chronic myeloid leukemia. Int J Hematol. 101:154-164, 2015.

**Table 1 ELN definitions of the response to TKIs, second line**

| Time | Optimal | Warning | Failure |
| --- | --- | --- | --- |
| Baseline | NA | High risk | NA |
|  |  | CHR never achieved |  |
|  |  | Loss of CHR |  |
|  |  | CyR never achieved |  |
|  |  | Mutations |  |
| 3 months | BCR-ABL1 ≤ 10 % or  Ph + <65 % | Ph + 65–95 % | No CHR |
|  |  |  | BCR-ABL1 > 10 % |
|  |  |  | Ph+ >95 % |
|  |  |  | New mutations |
| 6 months | BCR-ABL1 ≤ 10 % or  Ph + ≤ 35 % (MCyR) | Ph + 35–65 % | BCR-ABL1 > 10 % |
|  |  |  | Ph+ >65 % |
|  |  |  | New mutations |
| 12 months | BCR-ABL ≤1 % or  Ph + 0 (CCyR) | BCR-ABL1 1–10 % or  Ph + 1–35 % (PCyR) | BCR-ABL1 > 10 % |
|  |  |  | Ph+ >35 % |
|  |  |  | New mutations |

These definitions are provisional and mainly applicable to the results of second-line treatment with dasatinib and nilotinib

*NA* not applicable
